# Improved RNA preparation for RNA-seq of the intracellular bacterium *Wolbachia w*AlbB

**DOI:** 10.3389/fmicb.2025.1667452

**Published:** 2026-01-08

**Authors:** Lara V. Behrmann, Theresa A. Harbig, Achim Hoerauf, Kay Nieselt, Kenneth M. Pfarr

**Affiliations:** 1 Institute for Medical Microbiology, Immunology and Parasitology, University Hospital Bonn, Bonn, Germany; 2 Institute for Bioinformatics and Medical Informatics, University of Tübingen, Tübingen, Germany; 3 German Center for Infection Research (DZIF), Partner Site Bonn-Cologne, Bonn, Germany

**Keywords:** Dynabeads, endobacteria, mRNA depletion, riboPOOLs, RNA-seq, rRNA depletion, transcriptome analysis, *Wolbachia*

## Abstract

Despite advances in RNA-seq, investigating the transcriptome of intracellular bacteria remains challenging due to the substantial presence of host RNA. In the case of *Wolbachia* spp. that are propagated in insect cell lines, commercially available rRNA depletion kits are often not suitable. Here, we describe a method to study the transcriptome of *Wolbachia w*AlbB in the *Aedes albopictus* cell line C6/36. Custom-designed riboPOOLs (siTOOLs Biotech) were used to remove both prokaryotic and eukaryotic rRNA. To enrich the bacterial mRNA, eukaryotic mRNA was depleted using Dynabeads (Thermo Fisher Scientific). Compared to RNA prepared using the Illumina Ribo-Zero Plus Depletion Kit alone, additional depletion of eukaryotic mRNA increased wolbachial reads 7-fold to 0.7% of all reads. After removing eukaryotic and prokaryotic rRNAs with custom-designed riboPOOLs, there was a 300-fold increase of reads that mapped to *Wolbachia* (30.2%). Combining customized rRNA depletion from both organisms with eukaryotic mRNA depletion was more cost-effective than simply increasing the number of sequencing reads. This method can potentially be used for the enrichment of bacterial mRNA in studies of intracellular bacteria that cannot be propagated in standard cell lines.

## 1 Introduction

RNA sequencing (RNA-seq) has been used for almost 20 years for transcriptome analysis, offering unparalleled insights into the transcriptional landscape of cells and tissues. It is particularly useful for identifying differentially expressed genes, uncovering regulatory mechanisms, and understanding functional responses at the molecular level during processes such as development, disease, or treatment response (Wang et al., 2009; Stark et al., 2019).

Because ribosomal RNA (rRNA) accounts for the vast majority of total RNA, its presence can dominate sequencing reads, thereby limiting the detection of RNA species of interest, such as messenger RNA (mRNA) or non-coding RNAs. In eukaryotes, mRNA can be enriched via their poly(A) tails, whereas bacterial mRNAs lack this feature, making efficient rRNA depletion particularly critical. Commercially available RNA-seq kits are designed to streamline the process of rRNA depletion, library preparation, and sequencing, providing high-quality results for various biological systems. Standard kits are optimized for commonly used model organisms, i.e., human, mouse, rat, or bacteria (Kumar et al., 2012; Kumar et al., 2016; Koh et al., 2023; Cantin et al., 2024). However, when working with non-standard organisms, such as insect cell lines, the suitability of these kits can vary, especially for rRNA depletion. The differences in rRNA sequences in these species from those used to design commercial rRNA depletion probes prevent effective binding of the probes, resulting in insufficient rRNA removal and rRNA contamination that can mask the mRNA signal (Koh et al., 2023; Cantin et al., 2024). The challenge becomes even greater when studying intracellular bacteria, such as *Wolbachia,* in non-standard organisms or cell lines, as these bacteria often have small genomes and can be present in low numbers in host cells (Westermann et al., 2012; Raquin et al., 2015; Kumar et al., 2016). Consequently, transcriptome analysis of intracellular bacteria can be hampered by the presence of both bacterial and host rRNA, as well as host mRNA.

*Wolbachia* spp. are Gram-negative obligate intracellular Alphaproteobacteria found in arthropods and filarial nematodes. They are one of the most widespread endosymbionts, with 40–52% of arthropods being infected (Zug and Hammerstein, 2012; Weinert et al., 2015), and have medical relevance for hindering the transmission of viral diseases and treating diseases caused by filarial nematodes (Slatko et al., 2014). Nevertheless, analysis of the transcriptome of *Wolbachia* spp. is still difficult (Chung et al., 2020; Cantin et al., 2024).

While transcriptomics has been applied to study the effect of *Wolbachia* on their hosts, there are only a few studies on the transcriptomes of *Wolbachia* itself. There have been five studies on *Wolbachia* of the filarial nematodes *Onchocerca ochengi* (Darby et al., 2012), *Dirofilaria immitis* (Luck et al., 2014; Luck et al., 2015) and *Brugia malayi* (Grote et al., 2017; Chung et al., 2019), and five studies on *Wolbachia* of *Drosophila melanogaster* (Darby et al., 2014; Gutzwiller et al., 2015; Rainey et al., 2016; Lindsey et al., 2021) and *Aedes albopictus* (Leitner et al., 2021). Three of these investigated the effect of virus infection on *Wolbachia* (Rainey et al., 2016; Leitner et al., 2021; Lindsey et al., 2021). A recent re-analysis of the seven *Wolbachia* transcriptome data sets from non-viral studies revealed “a coordinated transcriptional response of translational proteins across diverse *Wolbachia* strains and host contexts,” although fewer than 100 differentially expressed genes were identified and a general lack of global gene regulation was concluded (Chung et al., 2020). The study also found that previous *Wolbachia* transcriptomic studies might not have achieved the necessary sequencing depth for differential expression analyses of *Wolbachia*, with usually less than 10% of all reads mapping to *Wolbachia*.

Although we recently published a cell-free system that allows for the extracellular cultivation of *Wolbachia w*AlbB for 12 days (Behrmann et al., 2024), long-term culturing of *Wolbachia* spp. is only possible *in vitro* in insect cell lines (Fallon, 2021). Several *Wolbachia*-infected insect cell lines have been established; primarily based on *A. albopictus* (e.g., C6/36 and Aa23) and *D. melanogaster* (JW18) (Fallon, 2021). This impedes their RNA analysis since insect cell rRNA depletion was less efficient with standard kits (Kumar et al., 2016). While rRNA depletion kits have been successfully developed for rRNA from the most commonly studied eukaryotes, i.e., human, mouse, rat, rRNA depletion kits for insect cells are limited to *Drosophila* (Kumar et al., 2012; Kumar et al., 2016; Koh et al., 2023; Cantin et al., 2024).

Here, we present a method based on custom-designed riboPOOLs and eukaryotic mRNA depletion, which resulted in 30.2% of reads mapping to the *Wolbachia w*AlbB genome. This method should enable further studies of the wolbachial transcriptome, including upon antibiotic treatment, and might be adapted for other intracellular bacteria and for viral studies.

## 2 Methods

### 2.1 C6/36 insect cell culture

The *A. albopictus* C6/36 insect cell line, uninfected or infected with the *Wolbachia pipientis* supergroup B strain of *A. albopictus* (*w*AlbB), was cultured as previously described (Turner et al., 2006; Henrichfreise et al., 2009). *Wolbachia*-infected C6/36 cells were incubated in plug-sealed 75 cm^2^ culture flasks (Greiner, Kremsmünster, Austria) at 26 °C in 15 mL Leibovitz’s L15 medium (Thermo Fisher Scientific, Waltham, Massachusetts, USA) supplemented with 20% heat-inactivated FCS (PAN-Biotech, Aidenbach, Germany), 1% MEM nonessential amino acids (Thermo Fisher Scientific), 2% tryptose phosphate broth (Sigma-Aldrich, St. Louis, Missouri, USA) and 1% penicillin/streptomycin (Thermo Fisher Scientific). They were passaged every 7 days, with 3 mL of the passage added to a new flask with 12 mL fresh medium. Medium exchange (10 mL) was performed 3 days after passaging.

For RNA isolation, insect cells were harvested using a cell lifter (Corning, New York, USA), stained with trypan blue (0.4%, Thermo Fisher Scientific), and counted in a Neubauer chamber (Laboroptik, Bad Homburg, Germany). The cells were then diluted to the desired concentration and either directly centrifuged or seeded into 12-well plates (Greiner) and further incubated in a final volume of 2 mL for up to 9 days [typical duration for antibiotic assays of *Wolbachia* (Johnston et al., 2014)]. Cells were harvested from the plates with a cell lifter (Sarstedt, Nümbrecht, Germany). Unless stated otherwise, cells were centrifuged at 500 g for 20 min at 4 °C (Centrifuge 5,417 R, Eppendorf, Hamburg, Germany), and the supernatant carefully removed via pipetting.

### 2.2 RNA preservation

Different insect cell numbers were tested, i.e., 6.75 × 10^6^, 10^5^, and 10^4^ cells/mL in duplicates. 200 μL cell culture of the different dilutions were harvested (160 g, 4 °C, 10 min), and various RNA preservation methods were tested: shock freezing in liquid nitrogen, RNAlater™ Stabilization Solution (Thermo Fisher Scientific), and QIAzol Lysis Reagent (Qiagen, Hilden, Germany).

For RNAlater preservation, cells were either stored in 50 μL RNAlater overnight at 4 °C, followed by supernatant removal via centrifugation (6,000 g, 4 °C, 10 min), and the pellet subsequently frozen at −80 °C (RNAlater I), or the cells were directly frozen in liquid nitrogen after the addition of RNAlater (RNAlater II). For QIAzol preservation, 700 μL of the reagent was added, the pellet was vortexed until fully suspended, and then frozen in liquid nitrogen. All samples were transferred from liquid nitrogen to −80 °C for storage until RNA extraction. Before extraction, RNAlater II samples were centrifuged (6,000 g, 4 °C, 10 min) and the RNAlater removed by pipette. 700 μL QIAzol was added to all pellets prior to the organic phase separation step.

In subsequent experiments, 1.8 mL of cell culture was harvested to simulate conditions suitable for drug susceptibility testing in 12-well plates, where a total volume of 2 mL is used, leaving 200 μL available for DNA extraction. In the last experiment with custom-designed riboPOOLs, 2 mL were harvested from cell culture flasks. The pellet was resuspended in 1 mL of QIAzol, an adaptation made to accommodate the increased starting material. The solution was then frozen in liquid nitrogen and stored at −80 °C until RNA extraction.

### 2.3 RNA extraction

All work surfaces were cleaned with RNase AWAY (Thermo Fisher Scientific), and pipettes, tips, and reagents were exclusively used for RNA assays to prevent contamination. An FFP2 mask was worn during all RNA handling steps to minimize the risk of contamination.

Total RNA was extracted from *w*AlbB-infected C6/36 cells using the miRNeasy Mini Kit (Qiagen, Hilden, Germany), with protocol adaptations. Samples were incubated at 37 °C in a heating block until completely thawed, ensuring that all salts were fully dissolved. After an additional 5 min incubation at room temperature, samples were mixed with 200 μL chloroform (Carl Roth, Karlsruhe, Germany) for the initial experiments or 100 μL 1-bromo-3-chloro-2-propanol (BCP, Tokyo Chemical Industry, Tokyo, Japan). BCP was preferred over chloroform due to its reduced toxicity and the need for only half the volume (Chomczynski and Mackey, 1995). The sample-chloroform/-BCP mixture was vigorously shaken for 15 s, followed by a 2–3 min incubation at room temperature. Phase separation was achieved through a 15 min centrifugation at 12,000 g, 4 °C. The upper aqueous phase, containing the nucleic acids, was retained, with 350 μL applied to the Qiagen column.

We included the optional DNase I treatment to eliminate contaminating DNA. Buffer RWT was prepared with isopropanol, as recommended by the manufacturer’s protocol for cases of low expected RNA yield. Column-based steps were performed manually for RNA preservation or automated using the QIAcube robotic workstation (Qiagen, Hilden, Germany) for subsequent experiments, according to the protocol. Total RNA was eluted in 30 μL of RNase-free water. 4 μL of RNA was set aside for further quality analysis, while the remaining RNA was stored at −80 °C.

### 2.4 Quality control

RNA concentration, A260/A280 and A260/A230 ratios were measured using the NanoVue spectrophotometer (VWR, Radnor, Pennsylvania, USA). RNA quality was assessed using the Experion Automated Electrophoresis Station (Bio-Rad, Hercules, California, USA). Based on the RNA concentrations determined by spectrophotometry, the RNA StdSens (700-7103, Bio-Rad) or HighSens (700-7105, Bio-Rad) kits were selected and used as per the manufacturer’s protocol. After each depletion step, NanoVue and Experion measurements were repeated to monitor any changes in RNA concentration and quality. The “scale to global” setting was used for all virtual gels to normalize the relative intensity of the lanes in the virtual gel.

To assess the efficacy of bacterial rRNA and eukaryotic mRNA depletion, RT-qPCRs were performed to quantify the copy number of wolbachial 16S rRNA and insect cell actin transcripts. No-RT controls were included to control for DNA contamination. cDNA synthesis was carried out using LunaScript RT SuperMix Kit (New England Biolabs, Ipswich, Massachusetts, USA) with 15 ng (initial testing of poly(A) depletion), 100 ng (initial testing of riboPOOLs and Terminator exonuclease), or 5 ng (depletion with custom-designed riboPOOLs) input RNA. Subsequent 16S rRNA and actin qPCRs were performed as previously described (Makepeace et al., 2006; Henrichfreise et al., 2009; Behrmann et al., 2024).

### 2.5 rRNA depletion

Different methods were tested for rRNA depletion. The Illumina Ribo-Zero Plus Depletion Kit (Illumina, San Diego, California, USA) was used to deplete rRNA from total RNA or poly(A)-depleted RNA, following the manufacturer’s protocol (see 2.7 rRNA sequencing).

The rRNA depletion using riboPOOLs (siTOOLs Biotech, Planegg, Germany) was carried out on total RNA according to the manufacturer’s instructions, using the maximum input of 5 μg RNA. Four different riboPOOLs were employed: Pan-Bacteria (dp-K012-26), *A. albopictus* (dp-K012-47), and custom-designed riboPOOLs for *Wolbachia* and *A. albopictus*. Initially, 1 μL of riboPOOLs was used per 20 μL reaction, as recommended by the manufacturer. Later, the riboPOOLs volume was increased to 2 μL (noted in relevant experiments), necessitating a proportional doubling of the streptavidin-coated magnetic beads. RNA clean-up was achieved through ethanol precipitation performed overnight at −20 °C, followed by washing steps the next day. When rRNA depletion with riboPOOLs was combined with eukaryotic mRNA depletion, ethanol precipitation was performed after the final depletion step.

In a third trial, the Terminator exonuclease (TER51020, Epicentre, Madison, Wisconsin, USA) was used for 2.5 μg of total RNA following the standard protocol, but without the addition of RNase inhibitor. The reaction was terminated using the riboPOOLs protocol.

### 2.6 Eukaryotic mRNA depletion

Eukaryotic mRNA was depleted using Dynabeads (Thermo Fisher Scientific), which have oligo (dT)_25_ residues that bind to the poly(A) tail of eukaryotic mRNA. The depletion generally followed the manufacturer’s instructions optimized for 75 μg of RNA in a volume of 100 μL with 100 μL binding buffer, with adaptations for RNA input in our study. In the initial Dynabeads test, 20–25 μL of total RNA were mixed with 25 μL binding buffer. For the Ribo-Zero and riboPOOLs comparison, 12 μg of total RNA or 0.25 μg of rRNA-depleted RNA were used per reaction for the first set of biological replicates, while 26–29 μg of total RNA or 0.3–0.6 μg of rRNA-depleted RNA were used per reaction for the second set. In these cases, 20 μL of total RNA were diluted with 80 μL H_2_O and mixed with 100 μL binding buffer; for rRNA-depleted RNA, 80 μL of RNA were mixed with 20 μL H_2_O and 100 μL binding buffer. For the final RNA-seq (RNA-seq run 3), 1.1–1.4 μg of rRNA-depleted RNA in a total volume of 170 μL were used, and, following the manufacturer’s instructions for dilute RNA, an equal volume of binding buffer was added. In all cases, the supernatant, containing RNA without eukaryotic mRNA, was not discarded but retained for further processing. The mRNA fraction bound to the beads was eluted in 5 μL of 10 mM Tris–HCl. Ethanol precipitation was performed as described earlier before samples were analyzed via Experion and some samples were sent for RNA-seq.

### 2.7 RNA sequencing

To determine the most effective strategy for RNA preparation, three sequencing runs were carried out on libraries generated with the different rRNA depletion methods.

RNA samples were quality checked and quantified using the Qubit RNA HS Assay Kit and an Agilent 2,100 Bioanalyzer using the RNA 6000 Pico Kit (Agilent). Library preparation was performed with the Illumina Stranded Total RNA Prep Kit, and ligation with the Illumina Ribo-Zero Plus Kit according to the manufacturer’s instructions without the initial rRNA depletion step for already rRNA-depleted samples. In brief, 5 ng of poly(A)-depleted RNA or rRNA- and poly(A)-depleted RNA was used for cDNA library construction, adapter ligation and 15 cycles of barcoding PCR using IDT for Illumina RNA UD Indexes Ligation. Obtained libraries were quantified with Qubit 1x DNA HS Assay Kit (Thermo Fisher) and the fragment distribution was checked on an Agilent 2,100 Bioanalyzer using High Sensitivity DNA Kit (Agilent).

The different rRNA depletion methods were compared with and without additional eukaryotic mRNA depletion using Dynabeads. Run 1 tested Ribo-Zero Plus depletion. Run 2 compared Ribo-Zero Plus and riboPOOLs depletion in combination with Dynabeads. Run 3 evaluated the performance of the custom-designed riboPOOLs. The first run included three libraries (biological replicates) and was sequenced as paired-end reads (100 bp) on a NovaSeq 6,000 platform (Illumina) to a depth of 28.9–34.6 million reads per library. The second run included four libraries corresponding to two technical replicates per condition and was sequenced as single-end reads (100 bp) on a MiSeq platform to a depth of 21–45 thousand reads per library. The third run included two technical replicates and was sequenced as single-end reads (100 bp) on a NovaSeq 6,000 platform (Illumina) to a depth of 178 and 197 million reads, respectively.

RNA sequencing of the first run was performed by the CeGaT GmbH, Tübingen. RNA sequencing of the second and third run was performed by the Institute for Medical Microbiology (part of the NGS Competence Center NCCT (Tübingen, Germany)) while data management for all runs, including storage of raw data for this project were done by the Quantitative Biology Center (QBiC, Tübingen, Germany).

For all three runs, sequencing statistics, including the quality per base and adapter content assessment of resulting transcriptome sequencing data were conducted with FastQC v0.11.5 (Andrews, 2015). All read mappings were performed independently against the reference strain of *Wolbachia pipientis w*AlbB (RefSeq ID: NZ_CP031221.1) and the reference strain of *A. albopictus* (RefSeq ID: GCF_006496715.1). The mappings of all samples were conducted with HISAT2 v2.1.0 (Kim et al., 2015). The first run with paired-end reads was run in paired-end mode with spliced alignment enabled. For the second and third runs, spliced alignment of reads was disabled and library type was set to reverse (HISAT2 parameter --no-spliced-alignment and --rna-strandness R).

The resulting mapping files in SAM format were converted to BAM format using SAMtools v1.9 (Li et al., 2009). Mapping statistics, including percentage of mapped reads and fraction exonic region coverage, were conducted with the RNA-Seq module of QualiMap2 v2.2.2-dev (Okonechnikov et al., 2016). Gene counts for all samples were computed with featureCounts v1.6.4 (Liao et al., 2014) based on the annotation of the respective reference genome, where the selected feature type was set to transcript records (featureCounts parameter -t transcript). A quality check for rRNA was performed with a self-written script based on the absolute counts of annotated rRNAs (Supplementary material - rRNA coordinates). For visualization of read coverage across rRNA loci, mapped reads were inspected using the Integrative Genomics Viewer (IGV, version 2.19.7) (Robinson et al., 2011).

All raw read datasets as well as count tables from featureCounts can be found in NCBI’s Gene Expression Omnibus under accession number GSE297421.

### 2.8 Design of riboPOOLs

To investigate the similarity of specific sequences to explicitly labeled rRNA genes, a multiple sequence alignment was created using MUSCLE (v.3.8) with default parameters (Edgar, 2004; Madeira et al., 2024) and a phylogenetic tree was created using iTOL (Letunic and Bork, 2024). Twelve sequences were identified with a similarity <95%, eight of which were already included in the *A. albopictus* riboPOOL. The remaining four were sent to siTOOLs Biotech and an additional riboPOOL was created. For *Wolbachia w*AlbB, the three rRNA sequences (5S, 16S, 23S) were sent to siTOOLs Biotech and a custom-designed riboPOOL created accordingly. This custom-designed riboPOOL has been added to the commercial repertoire of siTOOLs Biotech (dp-K012-86). The exact sequence of the riboPOOLs is proprietary knowledge of siTOOLs Biotech.

## 3 Results

### 3.1 Optimization of RNA preservation

**Figure 1:**
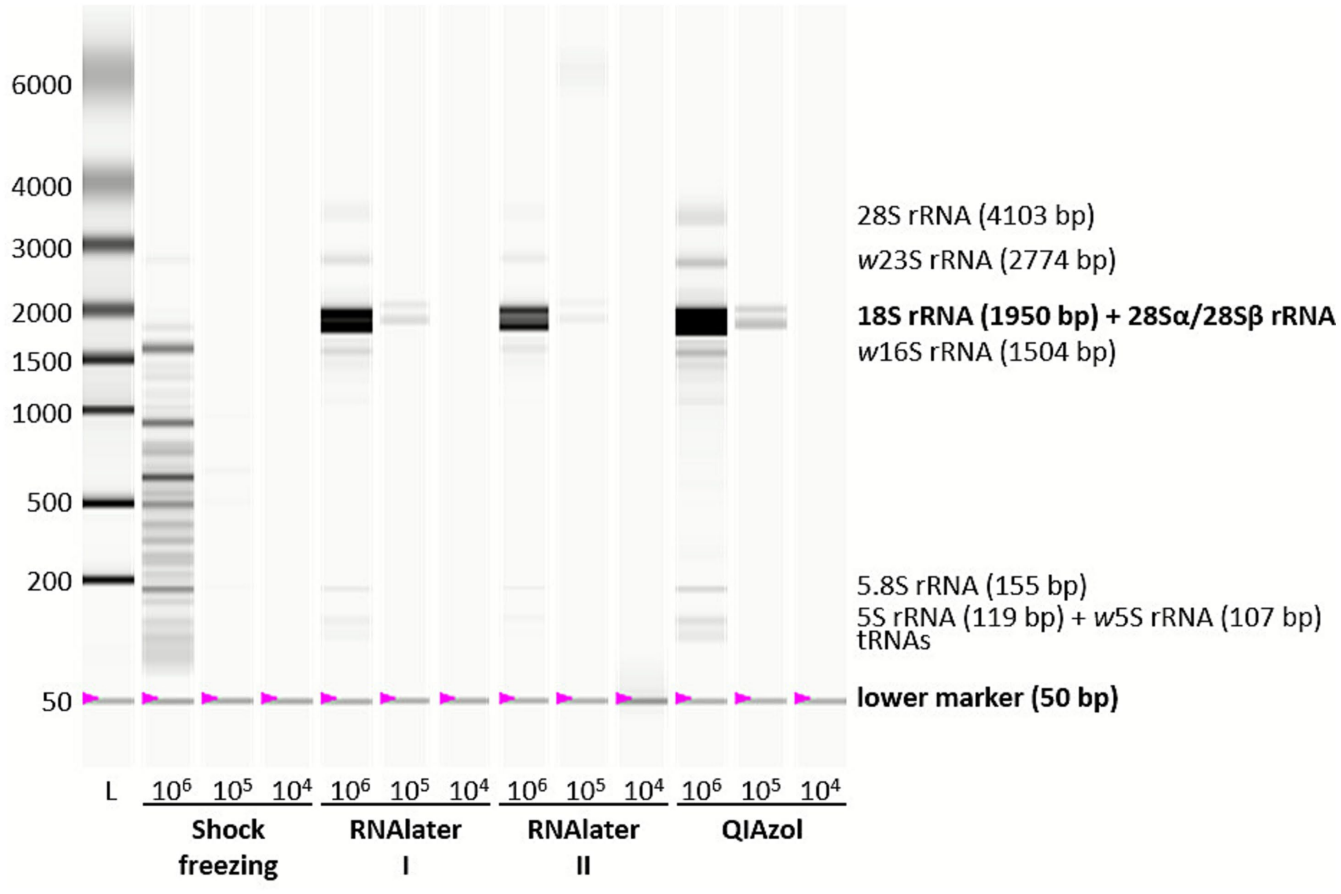
Virtual gel of RNA from different RNA preservation methods at varying insect cell concentrations. Insect cells, at concentrations of 6.75 × 10^6^, 10^5^, and 10^4^ cells/mL, were preserved using shock freezing, RNAlater (I: Incubation overnight at 4 °C with an additional washing step before freezing, II: Direct freezing), or QIAzol. RNA was extracted with the miRNeasy Mini Kit and subsequently analyzed with the Experion StdSens Kit using the eukaryotic total RNA protocol on the Experion Automated Electrophoresis Station. The resulting virtual gel is shown, with insect and *Wolbachia* rRNA as well as tRNA bands labeled.

Common issues in RNA extraction are low RNA yields and degradation. To optimize RNA preservation, different methods (shock freezing, RNAlater, and QIAzol) were tested at varying insect cell concentrations (6.75 × 10^6^, 10^5^, 10^4^ cells/mL). As expected, higher cell numbers resulted in increased RNA concentrations and better A260/A280 and A260/A230 ratios (data not shown). Further analysis with the Experion Station revealed that, while shock freezing produced the highest RNA concentration, it led to significant RNA degradation, with an RNA quality indicator (RQI) of 1.9 (an RQI ≥ 7 is recommended for RNA-seq) and multiple bands on the virtual gel (Table 1; Figure 1; Supplementary Figure 1). Freezing the pellet of 200 μL confluent cell culture (~10^6^ cells) in QIAzol yielded the best results, providing an RQI of 10 and twice the RNA concentration compared to RNAlater samples. For all subsequent experiments, cells were preserved in QIAzol before RNA extraction.

**Table 1:** Experion analysis of RNA from different preservation methods at varying insect cell concentrations.

RNA preservation method	Insect cell concentration (× 6.75/mL)	Concentration (ng/μL)	RQI^*^
Shock freezing	10^6^	118.96	1.9
	10^5^	5.39	/
	10^4^	1.33	/
RNAlater I	10^6^	55.29	10.0
	10^5^	6.47	/
	10^4^	1.11	/
RNAlater II	10^6^	42.63	10.0
	10^5^	4.91	/
	10^4^	4.77	/
QIAzol	10^6^	114.63	10.0
	10^5^	8.57	/
	10^4^	1.61	/

* The determined RNA quality indicator (RQI) values are shown. A dash (/) indicates that RQI could not be determined due to insufficient RNA concentration. RQI values ≥7 were considered indicative of acceptable RNA quality for RNA-seq.

Large rRNAs, 18S and 28S in eukaryotes and 16S and 23S in prokaryotes, are commonly assessed by gel electrophoresis to evaluate RNA integrity, with two distinct rRNA bands indicating intact RNA. However, insect cell rRNA often appears degraded, showing only one prominent band. This phenomenon is due to a “hidden break” in the 28S rRNA, which causes it to split into two fragments of similar size that co-migrate with 18S rRNA during denaturation (Gould, 1967; Ishikawa and Newburgh, 1972; Winnebeck et al., 2010).

### 3.2 Enrichment for wolbachial reads via rRNA and eukaryotic mRNA depletion

Because no commercial rRNA depletion kit is available for RNA-seq of *Wolbachia*, we tested different rRNA depletion methods with and without eukaryotic mRNA depletion.

**Figure 2:**
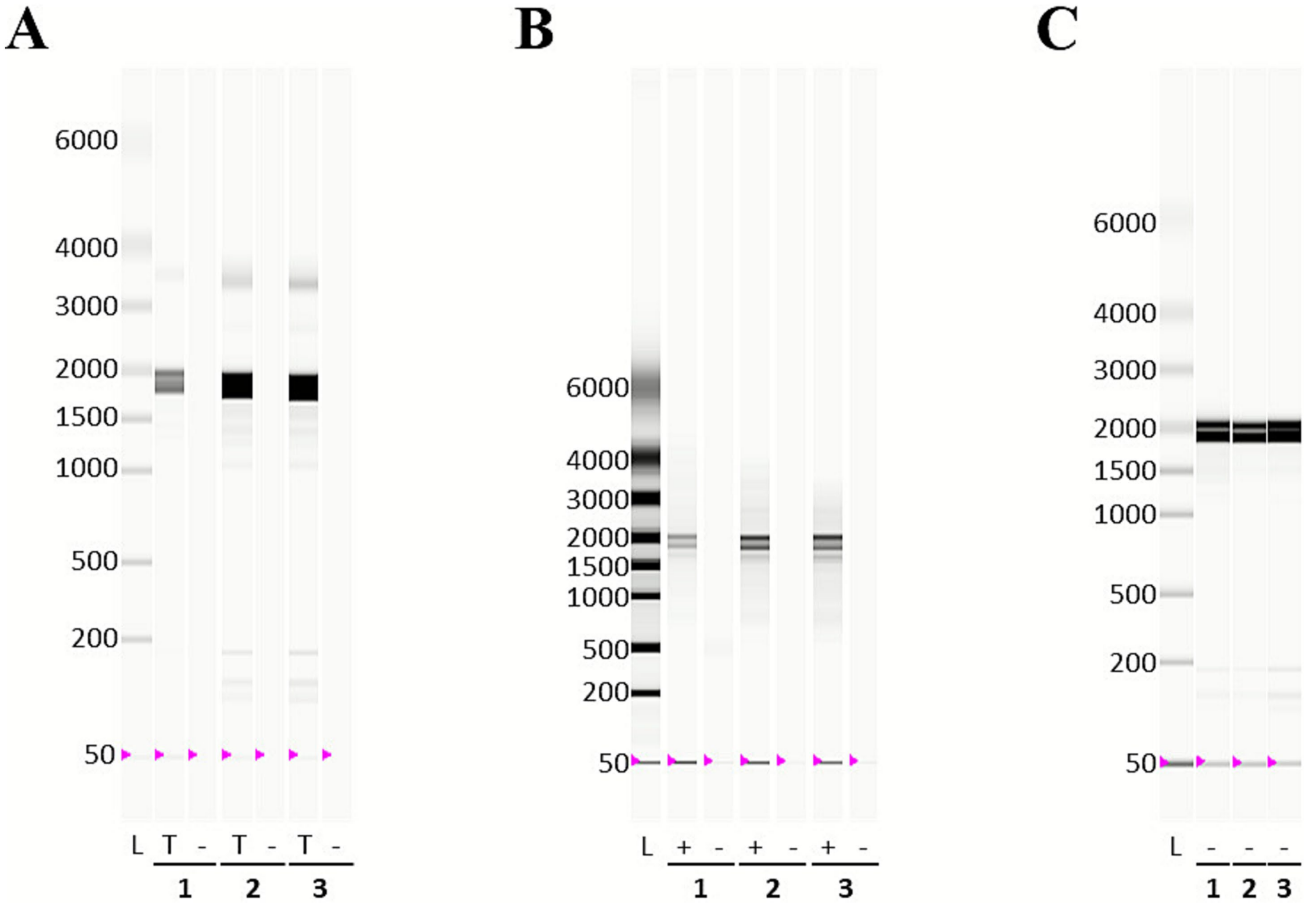
Virtual gels of RNA from poly(A) depletion. Total RNA was extracted from 0.5 × 10^7^ cells/mL cultured for 6 days (samples 1, 2) or 9 days (sample 3) in 12-well plates with the miRNeasy mini kit. Poly(A) depletion was performed and the resulting poly(A)-depleted RNA and eukaryotic mRNA were recovered. The different types of RNA were analyzed on the Experion Automated Electrophoresis Station and the virtual gels are shown. **(A)** Total RNA (T) and poly(A)-depleted RNA (−) were analyzed with the Experion StdSens Kit using the eukaryotic total RNA protocol. The total RNA of samples 1 and 3 was diluted 1:1 before measuring. **(B)** Eukaryotic mRNA (+) and poly(A)-depleted RNA (−) were analyzed with the Experion StdSens Kit using the mRNA protocol. **(C)** Poly(A)-depleted RNA (−) was diluted 1:100 and analyzed with the Experion HighSens kit using the eukaryotic total RNA protocol.

In our first trial, total RNA with excellent quality (RQI of 10, Supplementary Figure 2) was subjected to rRNA depletion with the Illumina Ribo-Zero Plus Depletion Kit. Of the total sequencing reads, 88.8% mapped to *A. albopictus* and only 0.1% mapped to *Wolbachia*. Of the respective mapped reads, 70.3% were *A. albopictus* rRNA and 22% were *Wolbachia* rRNA, clearly indicating that the rRNA depletion was inefficient. Due to the overwhelming abundance of eukaryotic mRNA compared to bacterial mRNA, we opted for using Dynabeads for poly(A) depletion rather than eukaryotic mRNA enrichment, as previously described (Kumar et al., 2016). We first evaluated the impact of poly(A) depletion on RNA integrity and quantity. We recovered 56% of poly(A)-depleted RNA from total RNA, with excellent A260/A280 and A260/A230 ratios (Table 2). Due to high salt concentrations and EDTA in the Dynabeads binding buffer, direct analysis via Experion was not possible (Figures 2A,B; Supplementary Figure 3A). After diluting the sample, Experion analysis was possible and an RQI of 10 was determined (Figure 2C; Table 2). cDNA synthesis followed by actin qPCR confirmed a 77% reduction in actin transcripts. 16S rRNA transcripts were found to increase to 150%, likely attributable to the effective reduction of eukaryotic transcripts, thus increasing the relative proportion of wolbachial transcripts (Supplementary Figure 3B).

**Table 2:** RNA quality analysis after poly(A) depletion.

Sample		c (ng/μL)	% of total RNA	RQI	A260/A280^a^	A260/A230^a^
1	Total RNA^b^	462.0	100	10	1.99	2.39
	Poly(A)-depleted^c^	157.5	76.71	10	2.04	2.19
	Eukaryotic mRNA^d^	15.5	0.84	-	2.07	0.98
2	Total RNA	468.0	100	10	2.07	2.43
	Poly(A)-depleted	129.0	55.13	10	2.04	2.31
	Eukaryotic mRNA	22.9	0.98	-	2.07	1.36
3	Total RNA	1173.2	100	10	2.07	2.35
	Poly(A)-depleted	190.7	36.56	10	2.04	2.17
	Eukaryotic mRNA	21.9	0.47	-	2.13	1.47

a The A260/A280 and A260/A230 ratios were determined with a NanoVue spectrophotometer.

b Total RNA was extracted from 0.5 × 10^7^ cells/mL cultured for 6 days (samples 1, 2) or 9 days (sample 3) in 12-well plates with the miRNeasy Mini Kit. Poly(A) depletion was performed and the resulting poly(A)-depleted RNA and eukaryotic mRNA were recovered. Total RNA was analyzed with the Experion StdSens Kit using the eukaryotic total RNA protocol on the Experion Automated Electrophoresis Station. The total RNA of samples 1 and 3 was diluted 1:1 before measuring.

c Poly(A)-depleted RNA (1:100 diluted) was analyzed with the HighSens Kit using the eukaryotic total RNA protocol on the Experion Automated Electrophoresis Station.

d Eukaryotic mRNA was analyzed with the Experion StdSens Kit using the eukaryotic total RNA protocol on the Experion Automated Electrophoresis Station. A hyphen (−) indicates that RQI was not determined due to selection of the mRNA protocol.

Analysis of the mRNA fraction showed that capturing of the eukaryotic mRNA was successful, resulting in 0.8% mRNA from total RNA, based on RNA amounts. According to Experion measurements, rRNA contamination was 10.1%, and faint rRNA bands were visible in the mRNA fractions, consistent with these measurements. Actin transcripts were enriched 30-fold in mRNA compared to total RNA.

**Figure 3:**
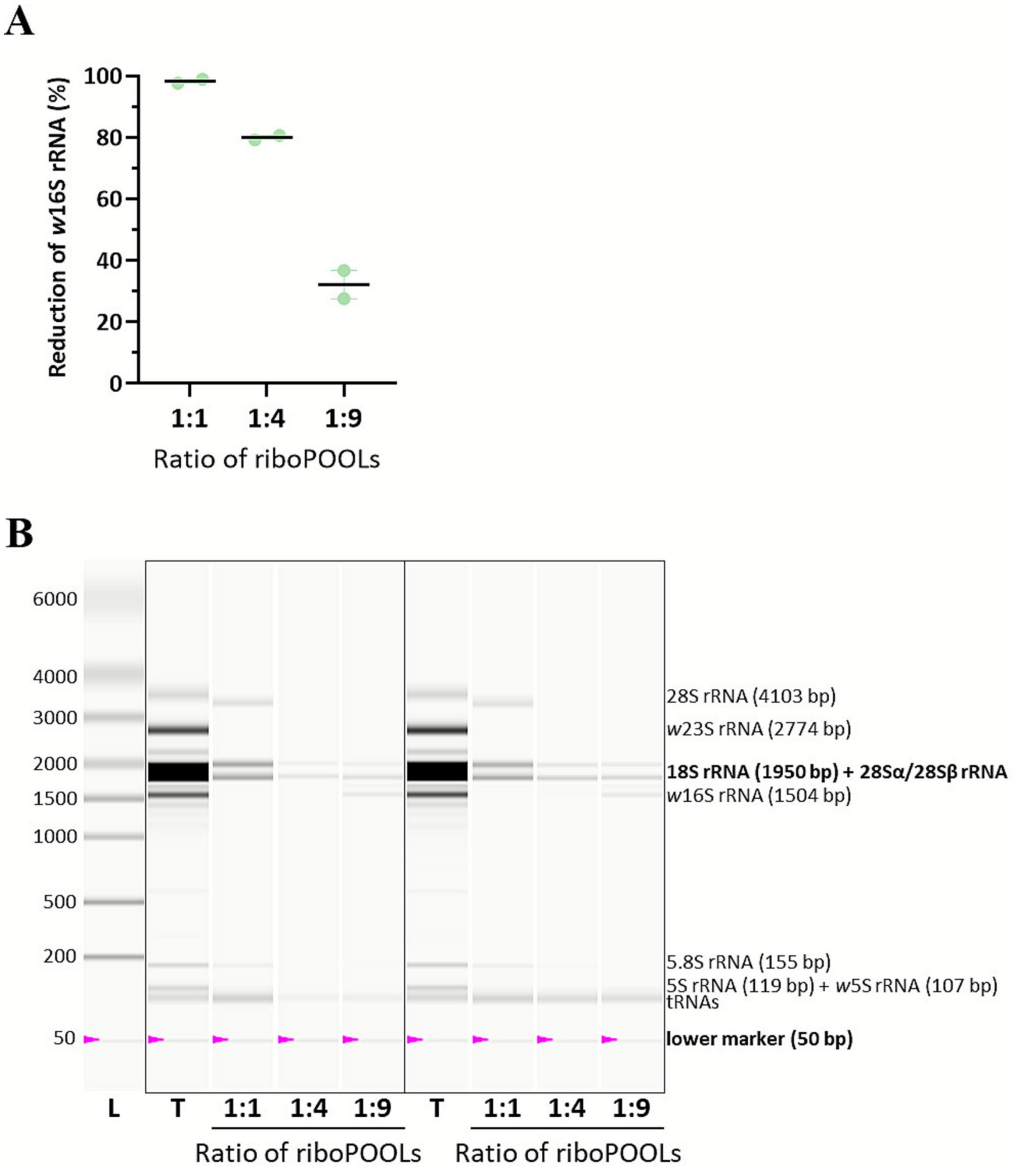
Ratio-dependent depletion of *Wolbachia* and insect cell rRNA using riboPOOLs. Total RNA was extracted with the miRNeasy mini kit in technical duplicates and subsequently treated with different ratios of pan-bacteria to *Aedes albopictus* riboPOOL (1:1, 1:4, 1:9). **(A)** *Wolbachia* 16S rRNA (*w*16S rRNA) copies were quantified via RT-qPCR. The reduction was calculated compared to the total RNA. The mean ± SEM is shown. **(B)** Total RNA (T) and RNA after treatment with different ratios of riboPOOLs were analyzed with the Experion StdSens Kit using the eukaryotic total RNA protocol on the Experion Automated Electrophoresis Station. The resulting virtual gel is shown, with insect and *Wolbachia* rRNA as well as tRNA bands labeled.

Next, we tested riboPOOLs that were designed for bacterial and *A. albopictus* rRNA depletion. Different ratios of the Pan-bacteria and *A. albopictus* riboPOOLs were tested and their efficiency assessed via 16S rRNA RT-qPCR (Figure 3A) and Experion analysis (Figure 3B; Supplementary Figure 4). A 1:4 ratio of Pan-bacteria to *A. albopictus* riboPOOL was found to lead to the best depletion. As expected, a 1:1 ratio showed stronger depletion of the wolbachial 16S rRNA (98%), while 1:4 and 1:9 ratios led to more depletion of the insect cell rRNA as visible from weaker bands on the virtual gel, with RNA concentrations equaling 9 and 10% of total RNA, respectively. Due to substantial 16S rRNA detected in the 1:9 samples via RT-qPCR (only 32% reduction) and the virtual gel, the 1:4 ratio was chosen. Integration of the 5S, 18S, and 28S rRNA peaks and normalization to RNA concentration determined an rRNA content of 61% in samples with a 1:4 ratio.

rRNA depletion with Terminator exonuclease was tested as well, however RNA concentrations were so low that we did not continue with this approach.

**Figure 4:**
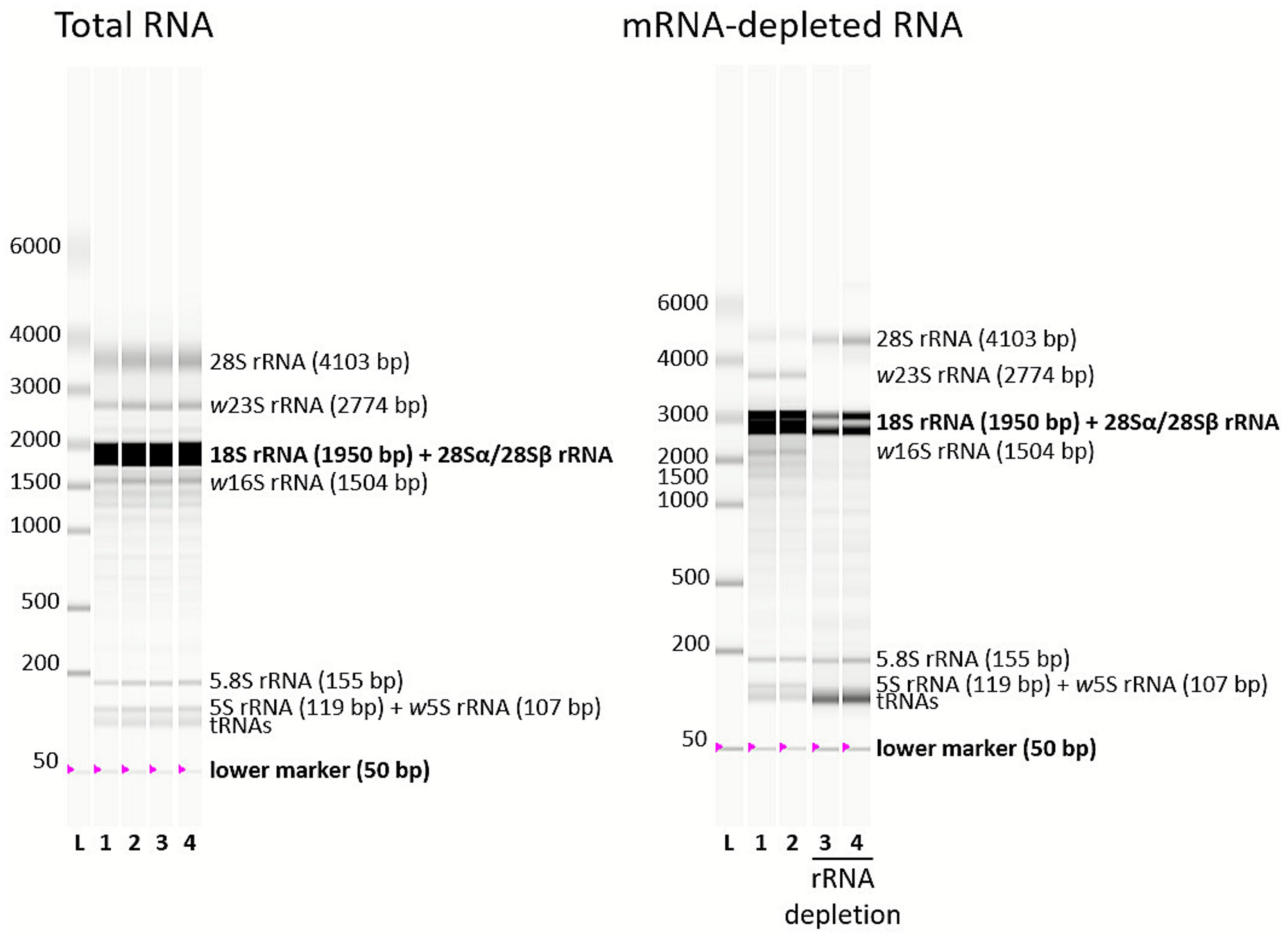
Virtual gels of poly(A)-depleted and rRNA- and poly(A)-depleted RNA. Total RNA was extracted with the miRNeasy Mini Kit in 4 technical replicates. Poly(A) depletion was performed with Dynabeads. rRNA depletion was performed using a 1:4 ratio of pan-bacteria to *Aedes albopictus* riboPOOL. Afterwards, poly(A) depletion was performed with Dynabeads. All RNA types were subsequently analyzed on the Experion Automated Electrophoresis Station. Total RNA was analyzed with the Experion StdSens Kit using the eukaryotic total RNA protocol, while the poly(A)-depleted RNA and rRNA- and poly(A)-depleted RNA were analyzed with the Experion HighSens Kit using the eukaryotic total RNA protocol. The poly(A)-depleted RNA was diluted 1:500 and the rRNA- and poly(A)-depleted RNA was diluted 1:10. The resulting virtual gels are shown, with insect and *Wolbachia* rRNA as well as tRNA bands labeled. The depleted samples were sent for RNA-seq.

As poly(A) depletion was successful, we then combined it with rRNA depletion either with the Ribo-Zero Plus Depletion Kit or with riboPOOLs. We performed biological duplicates with technical duplicates for each condition. The electropherograms show that rRNA depletion reduced the rRNA peaks (Supplementary Figure 5). The samples of the second biological replicate (virtual gels in Figure 4) were then RNA-sequenced.

Eukaryotic mRNA depletion in combination with Ribo-Zero Plus led to increased rRNA reads (83.9% for *A. albopictus*, 37.9% for *Wolbachia*) compared to Ribo-Zero Plus alone. Still, the percentage of reads mapping to *A. albopictus* was successfully decreased and the wolbachial reads increased to 0.7%, representing a 7-fold increase.

For the combination with riboPOOLs, despite the use of an *A. albopictus*-specific riboPOOL, rRNA reads constituted 92.2% for *A. albopictus*, higher than for Ribo-Zero Plus. The majority of the reads mapped to genes considered pseudogenes of *A. albopictus* in the genome annotation. Yet, the free text annotation description stated that the genes are rRNA genes (Supplementary material - rRNA coordinates). To investigate the similarity of these sequences to explicitly labeled rRNA genes, we created a multiple sequence alignment using MUSCLE (Edgar, 2004; Madeira et al., 2024). The pseudogenes showed high similarity to labeled rRNA genes (Supplementary Figure 6). Therefore, they were suspected to be rRNA genes and included into the estimation of the rRNA content. Nonetheless, wolbachial rRNA reads were reduced and with 0.8%, a slightly higher amount of wolbachial reads was achieved. This experiment confirmed the effectiveness of poly(A) depletion.

### 3.3 Custom-designed riboPOOLs

Closer examination revealed that the pseudogenes suspected to encode rRNA form two distinct clusters in the phylogenetic tree (Supplementary Figure 6). To reduce the number of genes, we filtered for sequences with less than 95% identity, which resulted in 12 distinct sequences. siTOOLs Biotech provided information on sequences targeted by their *A. albopictus* riboPOOL without mismatch, revealing that 8 of the 12 sequences were already targeted. We confirmed that no reads mapped to these eight sequences, indicating effective depletion.

The four remaining, untargeted sequences included two pseudogenes for the large subunit, one pseudogene for the small subunit, and one gene for 5.8S rRNA. Analysis of the RNA-seq reads revealed that these untargeted sequences accounted for >85% of all *A. albopictus* reads. We sent the untargeted sequences to siTOOLs Biotech, who then developed a pseudogene riboPOOL.

Additionally, a custom-designed riboPOOL was created for *Wolbachia* to improve rRNA depletion. The new riboPOOL efficiently depleted 16S rRNA in a test extraction as confirmed by the absence of the 16S rRNA peak in the electropherograms (Supplementary Figure 7). For a combination with the original *A. albopictus* riboPOOL, the total amount of riboPOOLs was increased from 1 to 2 μL. Since this led to smaller rRNA peaks in the electropherograms, the higher amount was used for the following experiments. However, we noted an increase for the putative peak for tRNAs.

**Figure 5:**
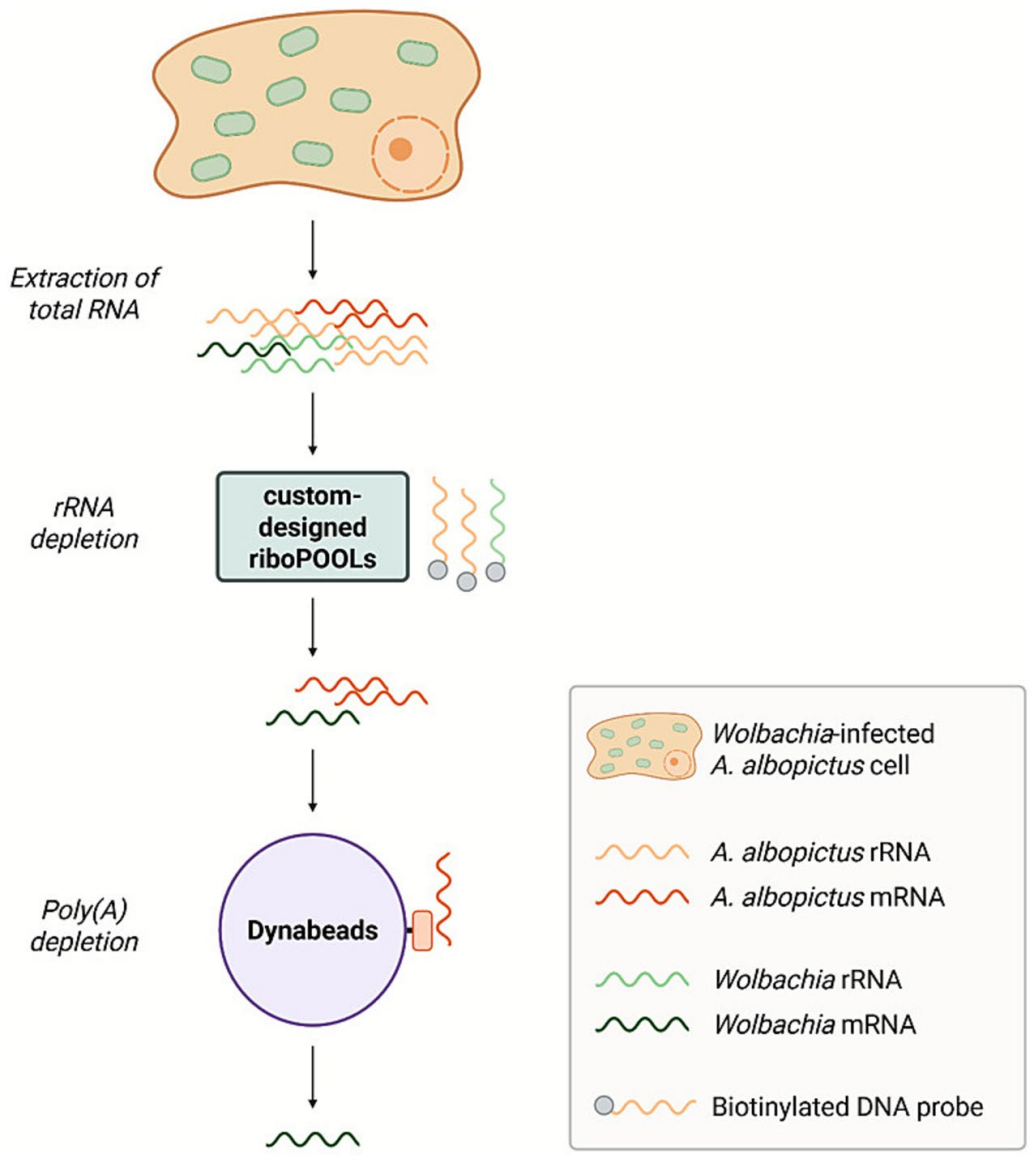
Schematic workflow of rRNA depletion with custom-designed riboPOOLs and poly(A) depletion. Total RNA is extracted from *Wolbachia*-infected *Aedes albopictus* cells. rRNAs are depleted using three custom-designed biotinylated DNA probe sets (riboPOOLs targeting *A. albopictus* rRNA, *A. albopictus* pseudo-rRNAs, and *Wolbachia* rRNA). Following rRNA removal, host mRNAs are captured via their poly(A) tails using Dynabeads coated with oligo(dT), resulting in a fraction enriched for *Wolbachia* mRNA. The schematic depicts the ideal depletion efficiency for both steps. Created in BioRender. Behrmann, L. (2026), https://BioRender.com/df2hy0s.

**Figure 6:**
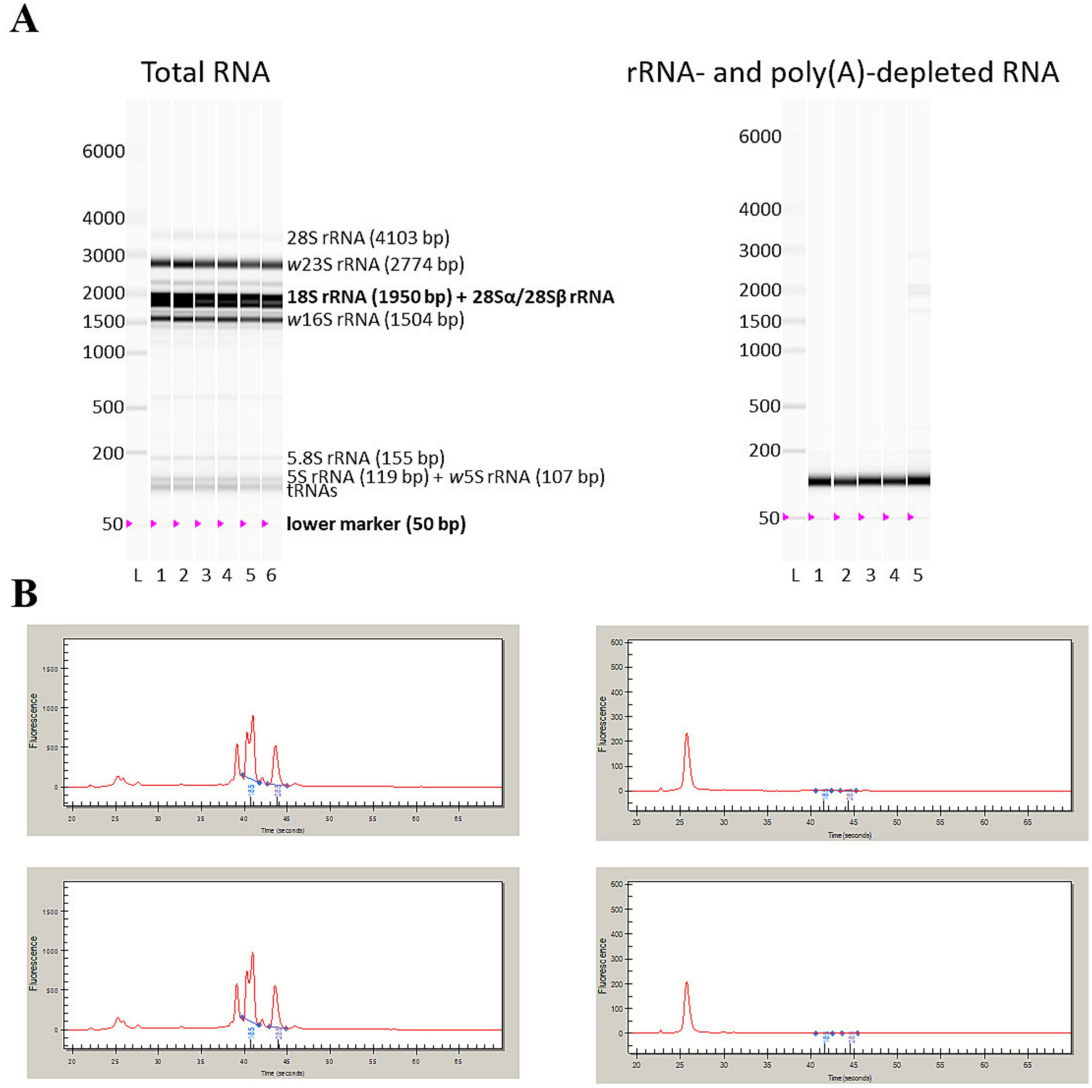
rRNA depletion with custom-designed riboPOOLs and poly(A) depletion. Total RNA was extracted with the miRNeasy mini kit in technical replicates, followed by rRNA depletion with riboPOOLs and subsequent poly(A) depletion. **(A)** Total RNA (six replicates) was analyzed with the Experion StdSens Kit, rRNA- and poly(A)-depleted RNA (five replicates) with the Experion HighSens kit, both using the eukaryotic total RNA protocol on the Experion Automated Electrophoresis Station. The resulting virtual gels are shown, with insect and *Wolbachia* rRNA as well as tRNA bands labeled. **(B)** Electropherograms before (left, total RNA) and after depletion (right, rRNA- and poly(A)-depleted RNA) for the two replicates (3, 4) that were RNA-sequenced. Note the different scales of the *y*-axis.

In the next attempt, the *Wolbachia* riboPOOL was combined with both *A. albopictus* riboPOOLs. The ratio of 1:4 was adapted to 3:8:4 for *Wolbachia*, *A. albopictus* and pseudogene riboPOOLs. The *Wolbachia* to total *A. albopictus* riboPOOL ratio was therefore still 1:4. rRNA depletion with these custom-designed riboPOOLs was combined with poly(A) depletion (Figure 5). RT-qPCR results were promising, with 96% 16S rRNA and 92% actin depletion (Supplementary Figure 8), indicating effective wolbachial rRNA and eukaryotic mRNA depletion. The virtual gel showed almost no bands for rRNA post-depletion (Figure 6A). The electropherograms of samples 3 and 4 are shown pre- and post-depletion (Figure 6B). The peaks for 5.8S, 16S, 18S, 23S, and 28S rRNA were completely absent after depletion. However, low molecular weight RNAs, likely 5S rRNA and tRNAs, were still present after depletion. It is important to note that the intensity of the bands cannot be compared between gels. The “scale to global” setting was used which means that the fluorescence value of the tallest peak from all electropherograms in one run is used to set the scale of the *y*-axis for all samples and thus the relative intensity of the lanes in the virtual gel. The intensity of the first peak was still increased for the depleted samples as can be seen from the electropherograms. This can again be due to the shift in composition of RNA-species as described earlier.

**Figure 7:**
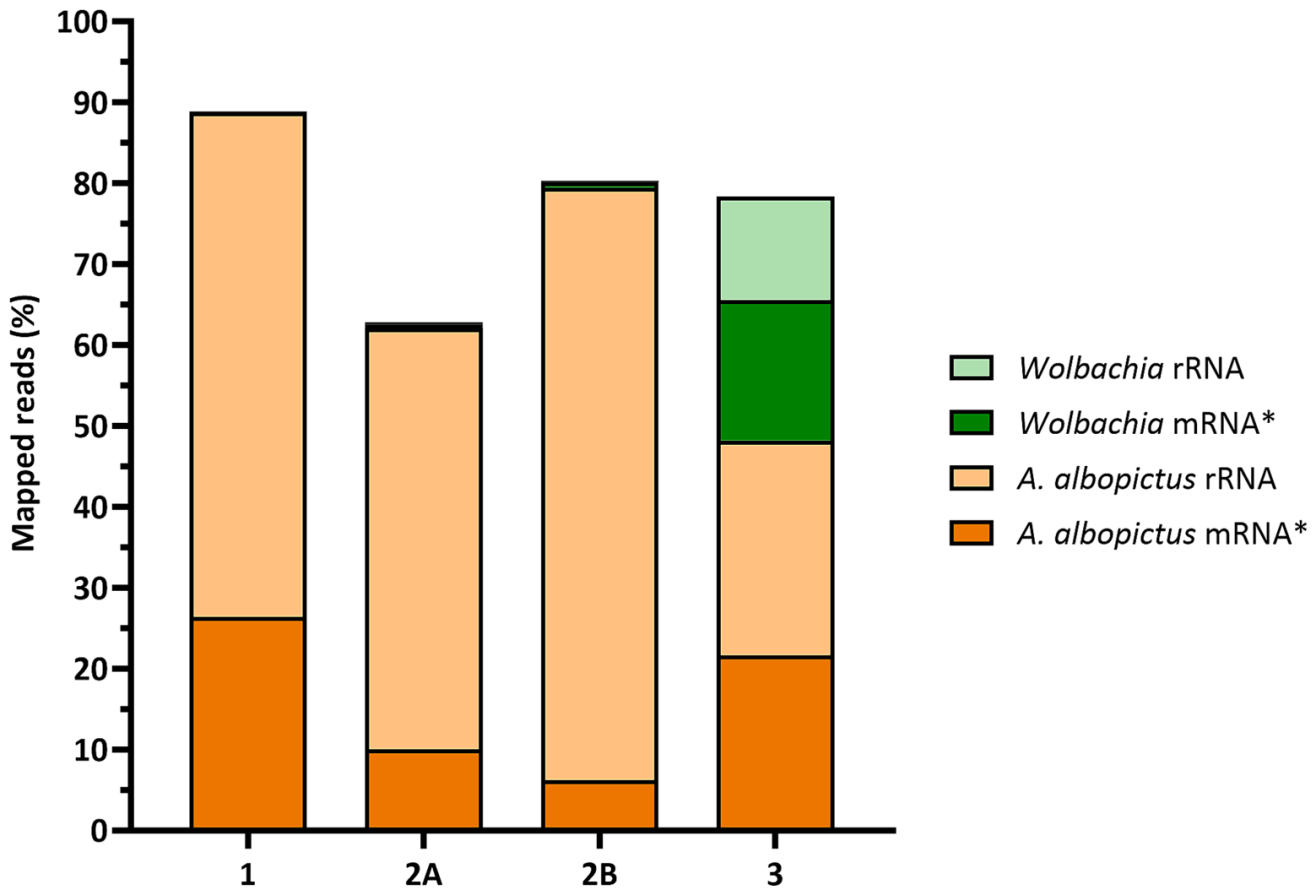
Mapped reads per sequencing run. Each column represents a sequencing run (run ID 1, 2A, 2B, 3). The height of each column reflects the percentage of reads that could be mapped in that run. Orange indicates *Aedes albopictus* reads, and green indicates *Wolbachia* reads. For each organism, darker shades correspond to mRNA* reads and lighter shades correspond to rRNA reads. *Annotated mRNA, tRNA, other non-coding RNAs.

This sequencing led to 48.2% of *A. albopictus* reads, of which 55.2% were rRNA. Still, the wolbachial reads were increased to 30.2%, with 42.5% rRNA. The median coverage was 166.5X, with 75.3% of the genome covered at ≥30X, ensuring sufficient depth for transcriptome analysis of *Wolbachia*. Table 3 summarizes the different RNA-seq runs in this study, and the mapped reads are visualized in Figure 7. Using IGV, the rRNA regions were visually inspected for both organisms. 28S rRNA of *A. albopictus* was almost completely depleted (Supplementary Figures 9A,B), consistent with the Experion analysis, whereas 5S rRNA remained highly covered (Supplementary Figure 9C). Similarly, 5S rRNA of *Wolbachia* showed higher coverage than 16S and 23S rRNA (Supplementary Figure 9D). When comparing protein-coding regions of *Wolbachia*, coverage was minimal in runs 1 and 2, but high in run 3 (Supplementary Figure 10).

**Table 3:** Overview of RNA-seq mapped reads after the different RNA preparation methods in this study.

		rRNA depletion^a^	rRNA + poly(A) depletion^b^		Custom-designed rRNA + poly(A) depletion^c^
run ID		1	2A	2B	3
rRNA depletion		Ribo-Zero Plus	Ribo-Zero Plus	riboPOOLs	Custom-designed riboPOOLs
Poly(A) depletion		-	Dynabeads	Dynabeads	Dynabeads
Mapped to *A. albopictus*	% of all reads	88.8 (88.0–89.6)	62.1 (61.5–62.6)	79.5 (78.3–80.7)	48.2 (48.0–48.4)
	% rRNA genes	70.3 (68.8–71.3)	83.9 (83.3–84.6)	92.2 (91.8–92.7)	55.2 (52.9–58.7)
Mapped to *w*AlbB	% of all reads	0.1 (0.1–0.1)	0.7 (0.7–0.7)	0.8 (0.8–0.8)	30.2 (29.9–30.4)
	% rRNA genes	22.0 (18.8–24.5)	37.9 (33.9–41.9)	17.3 (11.2–23.4)	42.5 (37.0–48.0)

a Run was performed in triplicate on a Flow Cell with the NovaSeq 6000 (28.9–34.6 million reads).

b Run was performed in duplicate for each combination on a Flow Cell (21–45 thousand reads).

c Run was performed in duplicate on a Flow Cell with the NovaSeq 6000 (178–197 million reads).

## 4 Discussion

We optimized sample preservation and RNA extraction to obtain high-quality RNA suitable for sequencing from *A. albopictus* cells. Using QIAzol and the miRNeasy Mini kit under these conditions, RNA integrity and concentration were consistently high (Table 1; Figure 1; Supplementary Figure 1). Further methodological details, including quality control procedures and alternatives to discontinued platforms, are provided in the Supplementary methods.

Our sequencing experiments employed three different rRNA depletion strategies, i.e., the Illumina Ribo-Zero Plus Depletion Kit, standard riboPOOLs (siTOOLs Biotech), and custom-designed riboPOOLs (siTOOLs Biotech). All of these methods were combined with poly(A) depletion with Dynabeads (Thermo Fisher Scientific), although Ribo-Zero Plus was tested without it in the first experiment.

The first RNA-seq (28.9–34.6 million total reads) led to only 0.1% reads mapped to *Wolbachia*. After performing an additional eukaryotic mRNA depletion with Dynabeads, we had 0.7% mapped to *Wolbachia* and 0.8% when we changed from the standard Ribo-Zero Plus rRNA depletion to specific riboPOOLs for Pan-Bacteria and *A. albopictus*. Although rRNA reads were increased for the riboPOOLs samples, wolbachial reads were also increased, confirming the efficiency of poly(A) depletion.

Pseudogenes of *A. albopictus* were identified as rRNA genes not targeted by the existing riboPOOLs. We therefore recommend checking for unannotated or pseudo-rRNA genes if rRNA depletion remains inefficient even after using specifically designed probes. Additionally, 5.8S rRNA was not efficiently removed. The design of a new riboPOOL for these genes as well as a *Wolbachia* riboPOOL and combining all three riboPOOLs led to 30.2% of reads mapping to *Wolbachia* in the third RNA-seq (178–197 million total reads).

Despite the almost complete removal of rRNA peaks in the Experion electropherograms for the samples depleted with custom-designed riboPOOLs, relatively high percentages of rRNA were detected in RNA-seq (Table 3). On the virtual gel (Figure 6A), bands corresponding to low-molecular weight RNAs were visible, and the electropherograms (Figure 6B) showed a prominent peak in this region. After rRNA depletion, low-molecular weight RNAs that were previously undetectable in total RNA could become visible due to the reduced dominance of rRNA, increasing the relative abundance of tRNAs and other small RNAs, and improving detection sensitivity. A similar observation was reported by Wahl et al. (2022) when using riboPOOLs for bacterial rRNA depletion. Many of the remaining reads in our dataset also mapped to a small nucleolar RNA (snoRNA) and a small nuclear RNA (snRNA). Furthermore, IGV analysis revealed that 5S rRNA from both *A. albopictus* and *Wolbachia* was not efficiently depleted. Still, it remains unclear why 55.2% rRNA reads for *A. albopictus* and 42.5% rRNA reads for *Wolbachia* remained, indicating that there is room for further optimization of the riboPOOLs or the rRNA depletion strategy, respectively. The difficulty in depleting 5S rRNA may be related to its secondary structure: while larger rRNAs have extensive loops and single-stranded regions, 5S rRNAs are mostly double-stranded, potentially limiting their accessibility to probes (Robertus et al., 1974). Potential improvements could include increasing the hybridization temperature above 68 °C to disrupt thermostable structures, increasing the proportion of 5S rRNA probes in the mix, or designing new probes that specifically account for the secondary structure of 5S rRNAs.

The custom-designed riboPOOLs led to a 38-fold increase in wolbachial reads compared to the standard riboPOOLs used in run 2, demonstrating their effectiveness in enriching bacterial RNA. Given this success, it should be tested whether Dynabeads may no longer be necessary. RNA is lost during poly(A) depletion (Table 2) and Dynabeads can potentially contaminate or inhibit downstream assays (RT-qPCRs, RNA-seq). They may also bind to prokaryotic RNA containing adenine-rich regions, potentially introducing a bias in the observed transcriptome. Omitting poly(A) depletion in future experiments would allow for dual RNA-seq, enabling the simultaneous sequencing of both bacterial and eukaryotic transcripts.

While the increased coverage of the wolbachial genome could be attributed to improved RNA enrichment, the overall number of reads was also 11-fold higher for the final samples that were rRNA-depleted with custom-designed riboPOOLs compared to the samples depleted with Ribo-Zero Plus. As a result, the general increase in read count likely contributed to the higher coverage and depth. Given the higher coverage, the number of reads can be reduced to lower RNA-seq costs. For bacteria, 5–10 million non-rRNA reads were found to yield sufficient sequencing depth, with 2–3 million being enough for biological replicates (Haas et al., 2012). To achieve 5 million non-rRNA reads with our method, 24 million reads would be needed, allowing for a 5- to 10-fold reduction in total RNA-seq reads.

Our custom-designed *Wolbachia w*AlbB riboPOOL has been added to the commercial repertoire of siTOOLs Biotech (dp-K012-86), the pseudogene riboPOOL is available on request. While the use of custom-designed probes offers significant advantages in enriching specific RNA, it also comes with limitations. Custom probes are tailored for specific species and are not universally applicable. For instance, Koh et al. (2023) found that when using probes designed for *Aedes aegypti* on other species like *A. albopictus* and Culicine and Anopheline mosquitoes, 46–94% of reads post-depletion were still ribosomal. Similarly, the two *A. albopictus* riboPOOLs might not be suitable for other mosquito species. Wolbachial rRNA might be more conserved and future studies will show whether our *Wolbachia w*AlbB riboPOOL is also effective for other *Wolbachia* subspecies.

To our knowledge, our study represents the first RNA-seq study of *Wolbachia w*AlbB in an *A. albopictus* cell line. To contextualize our work, we compiled a table summarizing other RNA-seq studies of *Wolbachia*, offering a comparison of RNA depletion methods and percentage of wolbachial reads (Table 4). One study investigating the effect of virus infection on *Wolbachia* did not give the percentage of wolbachial reads from all reads and was therefore excluded (Lindsey et al., 2021).

**Table 4:** Overview about RNA-seq studies of *Wolbachia* in chronological order.

*Wolbachia* strain	Wolbachial reads (%)^a^	rRNA depletion method	Poly(A) depletion	Study
*w*Oo	5	No	No	Darby et al. (2012)
*w*MelPop-CLA	6 (M)	Terminator exonuclease	No	Darby et al. (2014)
*w*Di	0.7 (M)	No	No	Luck et al. (2014)
*w*MelPop	3	No	No	Woolfit et al. (2015)
*w*MelCS	1			
*w*MelPop-CLA	18			
*w*MelPop-CLA	8	RiboMinus Eukaryote Kit + MicrobExpress bacterial mRNA Enrichment Kit		
purified *w*MelPop-CLA	83			
*w*Mel	1.6 (Mdn)	RiboMinus Eukaryote Kit	No	Gutzwiller et al. (2015)
*w*Di	9 (M)	No	No	Luck et al. (2015)
*w*Mel	4.9 (M)	Ribo-Zero Magnetic Gold Kit (Human/Mouse/Rat)^*^	No	Rainey et al. (2016)
*w*Ana	1.8	No	No	Kumar et al. (2016)
	1	Ribo-Zero Kits (Human/Mouse/Rat + Bacteria)^*^	Yes	
*w*Bm	1.5 (M)	No	No	Luck et al. (2017)
	1.4 (M)			
	6.3 (M)	Cappable-Seq^#^		
*w*Bm	3.0 (M)	Terminator exonuclease	No	Grote et al. (2017)
*w*Bm	0.7 (M)	Ribo-Zero Kits (Human/Mouse/Rat + Bacteria)^*^	Yes	Chung et al. (2019)
	2.5 (M)	Agilent Sure Select Kit^#^ (Mosquito)	No	
	24.0 (M)	Agilent Sure Select Kit^#^ (Gerbil)		
*w*AlbB	29.7 (M)	Ribo-Zero Magnetic Gold Kit (Human/Mouse/Rat)^*^	No	Leitner et al. (2021)
*w*AlbB	2	VAHTS Total RNA-seq (Human/Mouse/Rat) Library Prep Kit	No	Hussain et al. (2023)
*w*Bm	1	Custom filarial nematode probes	No	Cantin et al. (2024)
*w*AlbB	0.1 (M)	Ribo-Zero Plus Kit	No	This study
	0.7 (M)		Yes	
	0.8 (M)	riboPOOLs (Pan-Bacteria + *Aedes albopictus*)		
	30.2 (M)	custom riboPOOLs (*w*AlbB + *Aedes albopictus*)		

a If known, it is stated whether the mean (M) or median (Mdn) are given. The wolbachial reads were given or calculated based on data deposited in the Sequence Read Archive (Darby et al., 2014), data in the manuscript (Grote et al., 2017; Luck et al., 2017; Chung et al., 2019; Leitner et al., 2021), or data provided by the authors (Rainey et al., 2016).

* Discontinued kits are marked with an asterisk.

# Indirect rRNA depletion via enriching for mRNA.

While the percentage of mapped reads is an important metric, it must be interpreted with caution. Without knowing how many of these reads correspond to rRNA genes, this value alone may not provide a comprehensive understanding of genome coverage, e.g., Luck et al. (2014) mentioned that of wolbachial reads, >75% were rRNA reads. However, the amount of wolbachial rRNA reads was not given in each of these studies.

Previous methods for rRNA depletion in non-mammalian hosts relied on kits that are no longer available (see Supplementary methods for details). As a replacement, we tested the Ribo-Zero Plus Depletion Kit (Illumina) for human, mouse, rat, and bacteria, and found that depletion of insect rRNA was not efficient (Table 3). Bacterial rRNA depletion was more efficient and led to a reduction to 22%. This approach yielded only 0.1% wolbachial reads. Darby et al. (2014) and Grote et al. (2017) used Terminator exonuclease (Epicentre) for rRNA depletion and achieved a mean of 6 and 3% wolbachial reads, respectively. However, they did not state the remaining rRNA content. Cantin et al. (2024) also used Terminator exonuclease and achieved more than 3% wolbachial reads, almost all (>98%) originating from rRNA. For us, RNA concentration after Terminator exonuclease treatment was so low that an RQI could not be calculated, so we did not attempt RNA-seq.

In addition to the 10 studies of the wolbachial transcriptome, RNA-seq was applied in five other studies of *Wolbachia* of *D. melanogaster*, *Drosophila ananassae*, *B. malayi*, and *A. albopictus* (Woolfit et al., 2015; Kumar et al., 2016; Luck et al., 2017; Hussain et al., 2023; Cantin et al., 2024).

Woolfit et al. (2015) observed strong differences in wolbachial reads depending on sample type, with cell culture samples yielding the highest coverage of the *Wolbachia* genome. While RNA isolated from fly heads only led to 3% (*w*MelPop) and 1% (*w*MelCS) wolbachial reads, RNA from cell culture samples (*w*MelPop-CLA) led to 8% wolbachial reads when sequenced on GAII and to 18% when sequenced on HiSeq. Among these wolbachial reads, 94–96% corresponded to rRNA genes, except for the sample with 8% wolbachial reads, with a comparably low share of 63% rRNA reads. RNA-seq of RNA from purified *Wolbachia* (*w*MelPop-CLA) led to 83%, the highest percentage of wolbachial reads, with 92% of these reads mapping to *Wolbachia* rRNAs. However, it is unclear whether such a purification procedure affects the transcriptome.

Depending on study design and objectives, purification of *Wolbachia* might be a cost-efficient solution for RNA-seq. Where appropriate, our extracellular culture system of *Wolbachia* could be applied for this purpose (Behrmann et al., 2024), as purification is performed in the beginning, minimizing the risk of transcriptome alteration before harvest. However, this system has only been developed for the *w*AlbB strain.

Alternative methods for enriching *Wolbachia* mRNA are Cappable-seq and the Agilent SureSelect Kit (Luck et al., 2017; Chung et al., 2019). Cappable-seq, which captures 5′-triphosphorylated RNA, increased wolbachial reads by 4.2-fold compared to untreated total RNA but still yielded only 6.5% wolbachial reads (Luck et al., 2017). The Agilent SureSelect Kit, which uses sequence-specific hybridization probes to capture mRNA, achieved a relatively high proportion of wolbachial reads in gerbil samples (Chung et al., 2019). However, this approach introduces hybridization bias, meaning only pre-selected, well-annotated transcripts are efficiently captured, potentially missing novel or unannotated *Wolbachia* transcripts.

A recent study by Cantin et al. (2024) achieved 1% wolbachial reads for *w*Bm while employing dual RNA-seq. They investigated various rRNA depletion methods and found that DNA probes were most effective, consistent with our findings. Their depletion, however, was RNase H-based, whereas we used a bead-based depletion strategy (riboPOOLs). Importantly, their focus was on enabling dual RNA-seq of *B. malayi* female worms and their *Wolbachia*, while our study aimed to solely increase wolbachial read counts including through the depletion of eukaryotic mRNA. A future comparison of their RNase H-based approach – using DNA probes designed to target *A. albopictus* and wolbachial rRNA, including the *A. albopictus* rRNA pseudogenes described in this study – with our riboPOOL-based strategy would help identify whether enzymatic and bead-based depletion are equally suitable.

Using our custom-designed riboPOOLs in combination with eukaryotic mRNA depletion, we achieved a high level of wolbachial reads (30.2%), offering a powerful approach for RNA-seq studies of *Wolbachia*. We hope that our method for RNA preparation will enable transcriptomic analyses under diverse conditions to further elucidate gene regulation in this important endosymbiont, e.g., across host developmental stages, in different host tissues, or upon antibiotic treatment. Importantly, this approach can be adapted for other intracellular bacteria and extended to viral studies, thereby broadening its relevance for host-microbe research.

## Data Availability

The datasets presented in this study can be found in online repositories. The names of the repository/repositories and accession number(s) can be found in the article.
