# Supplementary material for: Improved RNA preparation for RNA-seq of the intracellular bacterium Wolbachia wAlbB
Source: Front Microbiol. 2026 Jan 8;16:1667452. doi: 10.3389/fmicb.2025.1667452 (PMC12865608; doi:10.3389/fmicb.2025.1667452)
Supplement: Supplementary file 1 [file Data_Sheet_1.PDF]

Supplementary Material

Supplementary Figures

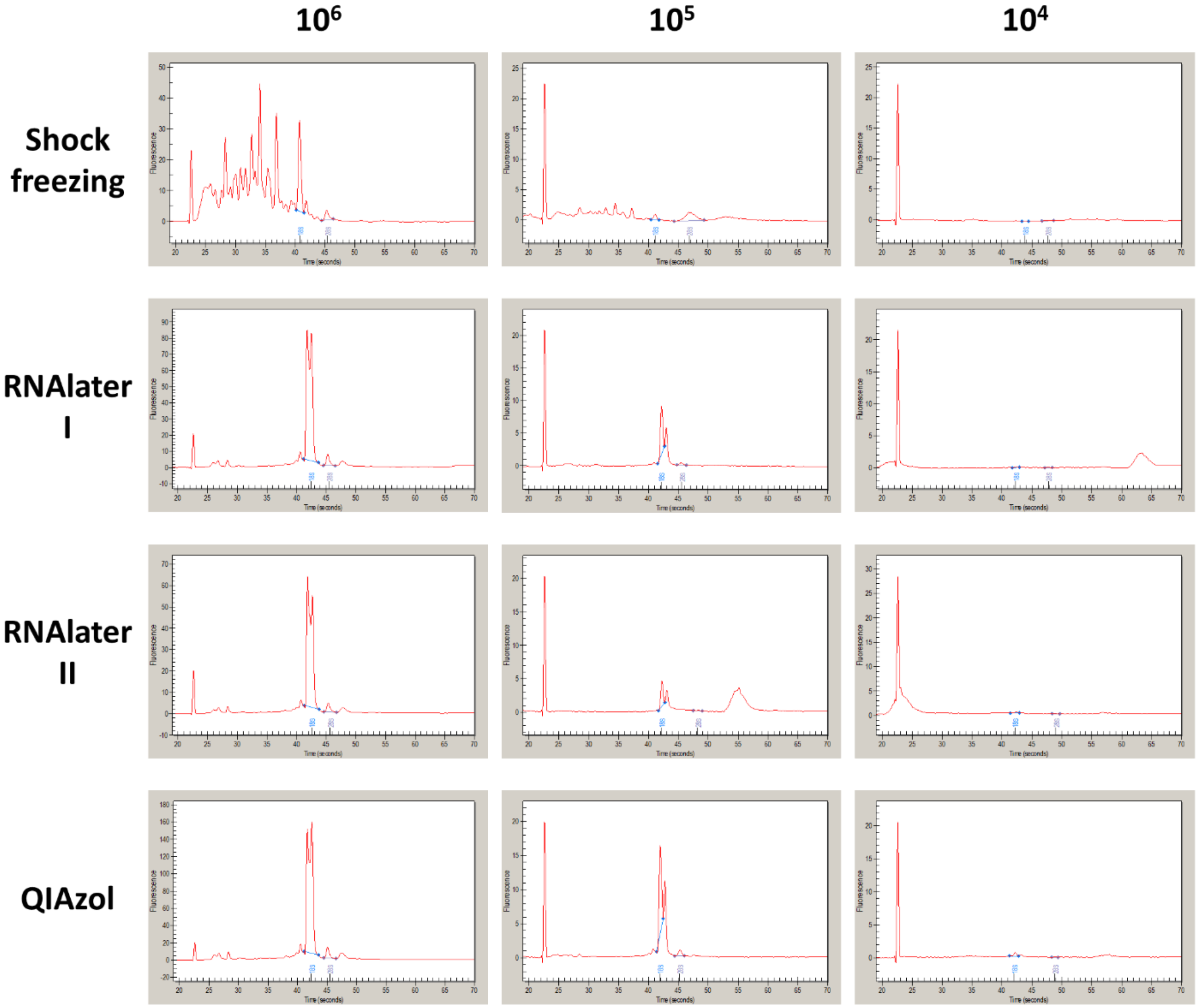

Supplementary Figure 1. Electropherograms of RNA from different preservation methods at varying insect cell concentrations. Insect cells, at concentrations of 6.75 × 10^6, 10^5, and 10^4 cells/ml, were preserved using different methods: shock freezing, RNAlater (I: incubation overnight at 4 °C with an additional washing step before freezing, II: direct freezing), or QIAzol. Total RNA was extracted with the miRNeasy Mini Kit and subsequently analyzed with the Experion StdSens Kit using the eukaryotic total RNA protocol on the Experion Automated Electrophoresis Station.

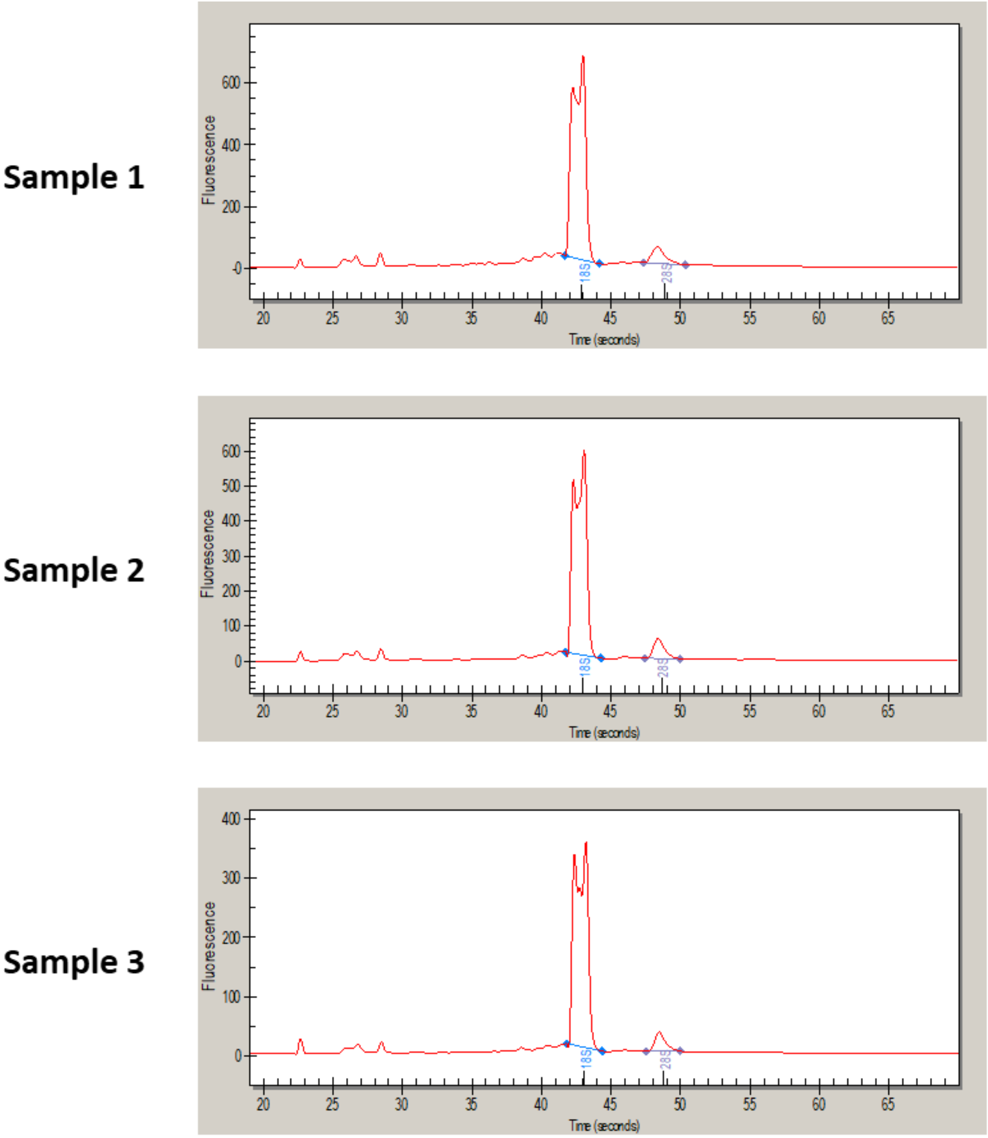

Supplementary Figure 2. Electropherograms of total RNA for RNA-seq. Insect cells were seeded at a density of 0.5×10^7 cells/ml (samples 1, 2) or 0.5×10^5 cells/ml (sample 3) and cultured for 6 days (samples 1, 2) or 9 days (sample 3) in 12-well plates. Total RNA was extracted with the miRNeasy Mini Kit. Analysis was performed with the Experion StdSens Kit using the eukaryotic total RNA protocol on the Experion Automated Electrophoresis Station. The samples were sent for RNA-seq.

A

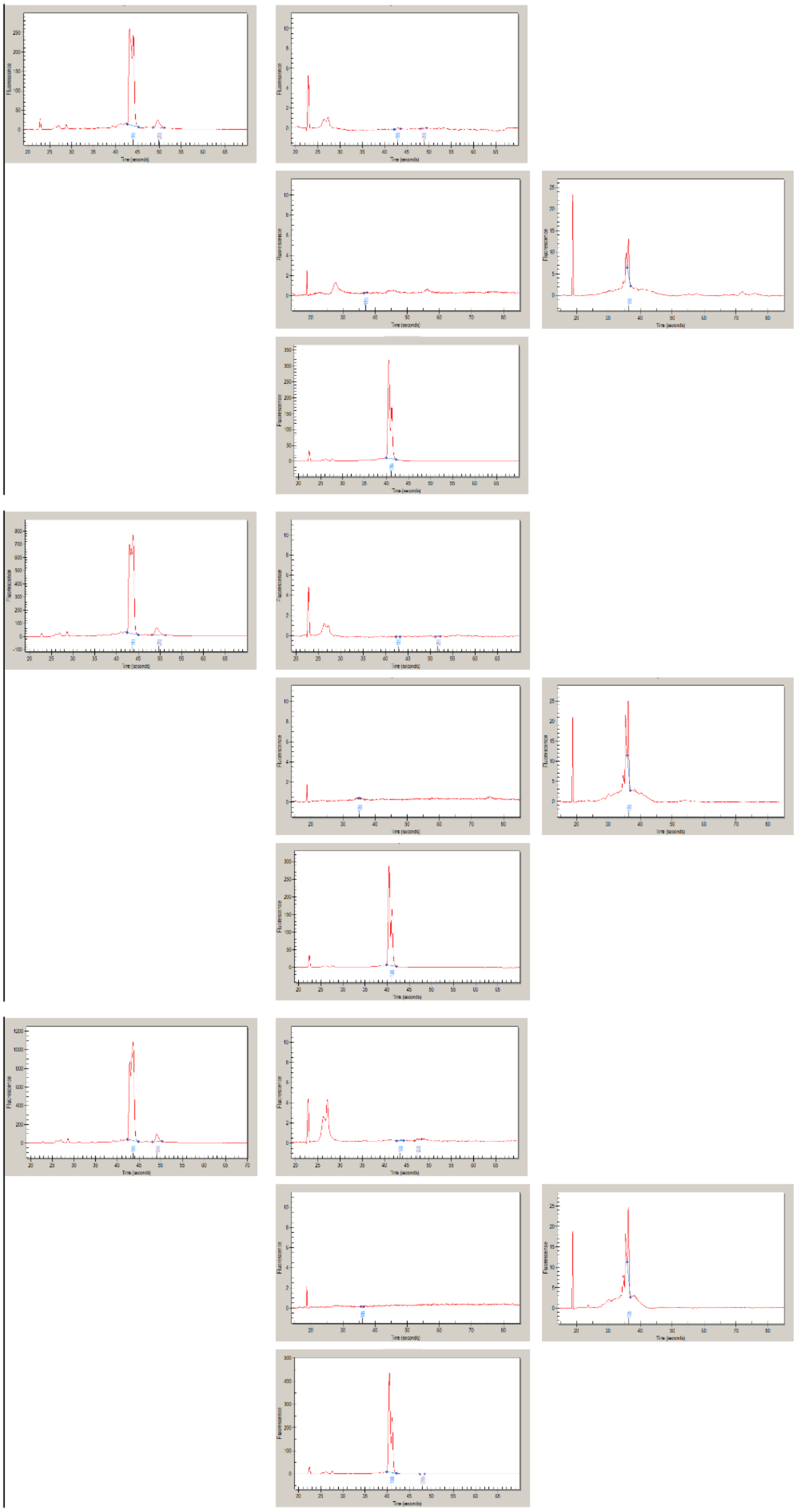
Total RNA
Poly(A)-depleted
Eukaryotic mRNA
Sample 1
Sample 2
Sample 3
Experion StdSens (eukaryotic total RNA protocol)
Experion StdSens (mRNA protocol)
Experion HighSens (eukaryotic total RNA protocol)
Experion StdSens (eukaryotic total RNA protocol)
Experion StdSens (mRNA protocol)
Experion HighSens (eukaryotic total RNA protocol)
Experion StdSens (eukaryotic total RNA protocol)
Experion StdSens (mRNA protocol)
Experion HighSens (eukaryotic total RNA protocol)

B

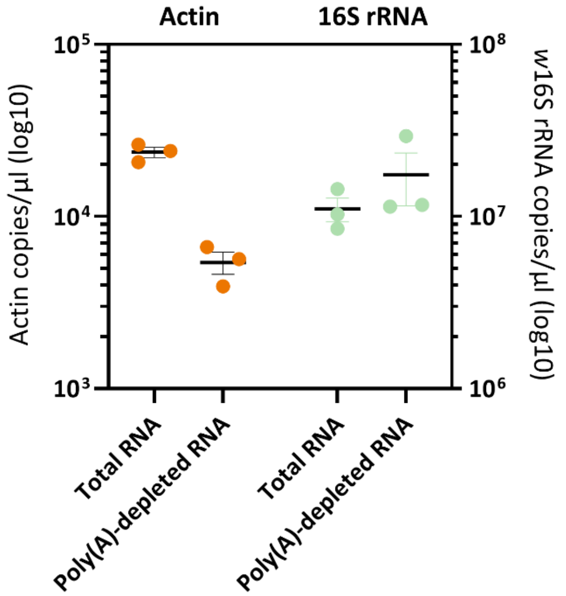

Supplementary Figure 3. Depletion of insect cell mRNA using Dynabeads. Total RNA was extracted from 0.5 × 10^7 cells/ml cultured for 6 days (samples 1, 2) or 9 days (sample 3) in 12-well plates with the miRNeasy Mini Kit. Poly(A) depletion was performed and the resulting poly(A)-depleted RNA and eukaryotic mRNA were recovered. The different types of RNA were analyzed on the Experion Automated Electrophoresis Station and by RT-qPCR. (**A**) Electropherograms of different analyses are shown. First, total RNA and poly(A)-depleted RNA were analyzed with the Experion StdSens Kit using the eukaryotic total RNA protocol. The total RNA of samples 1 and 3 was diluted 1:1 before measuring. Second, poly(A)-depleted RNA and eukaryotic mRNA were analyzed with the Experion StdSens Kit using the mRNA protocol. Third, poly(A)-depleted RNA diluted 1:100 was analyzed with the Experion HighSens Kit using the eukaryotic total RNA protocol. (**D**) Insect cell actin (orange) and wolbachial 16S rRNA (*w*16S rRNA, green) transcripts were quantified via RT-qPCR in total RNA and poly(A)-depleted RNA. The mean ± SEM is shown.

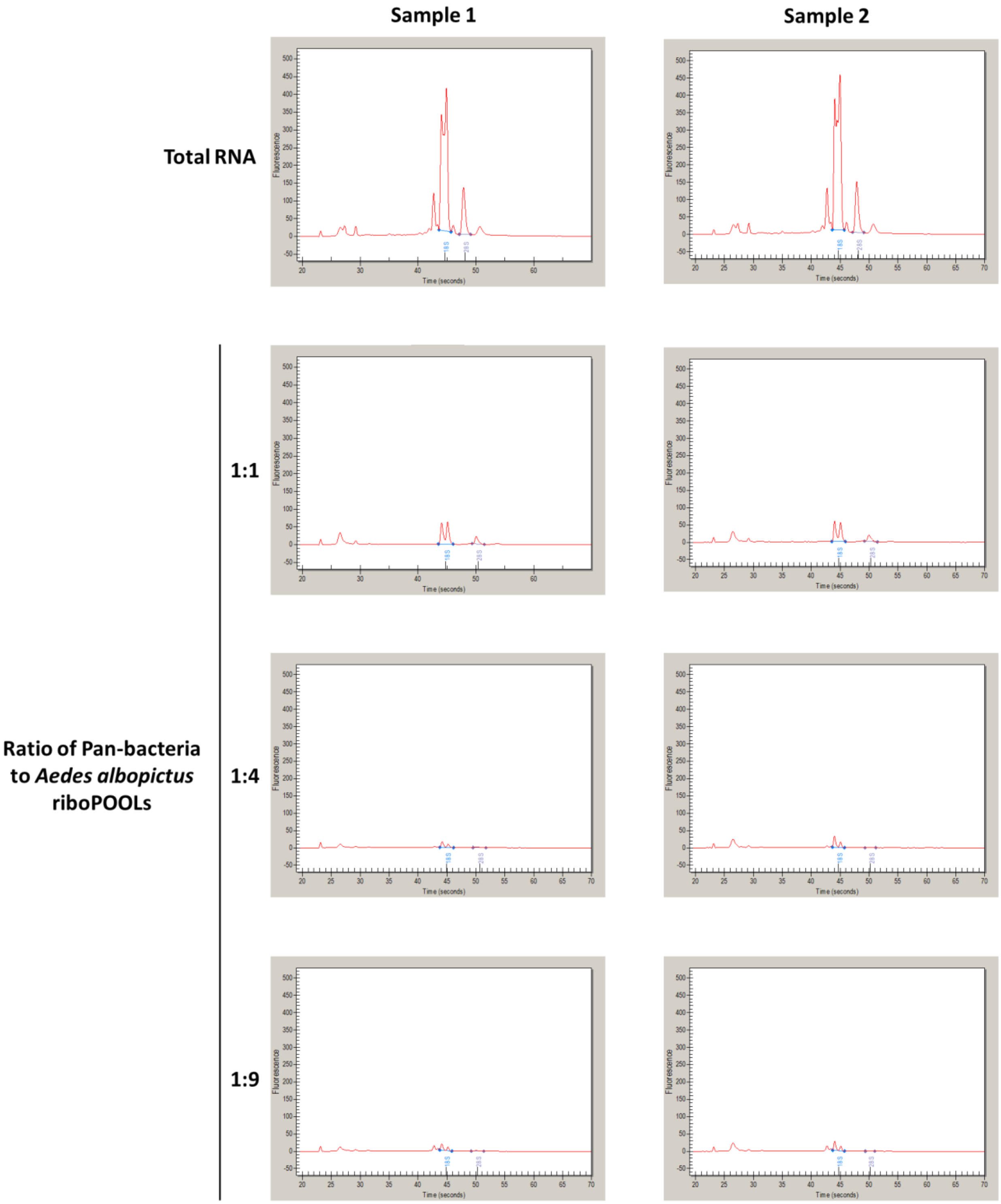

Supplementary Figure 4. Electropherograms showing ratio-dependent depletion of wolbachial and insect cell rRNA by riboPOOLs. Total RNA was extracted with the miRNeasy Mini Kit in technical duplicates and subsequently treated with different ratios of Pan-Bacteria to *Aedes albopictus* riboPOOLs (1:1, 1:4, 1:9) for rRNA depletion. Total RNA and rRNA-depleted RNA were analyzed with the Experion StdSens Kit using the eukaryotic total RNA protocol on the Experion Automated Electrophoresis Station.

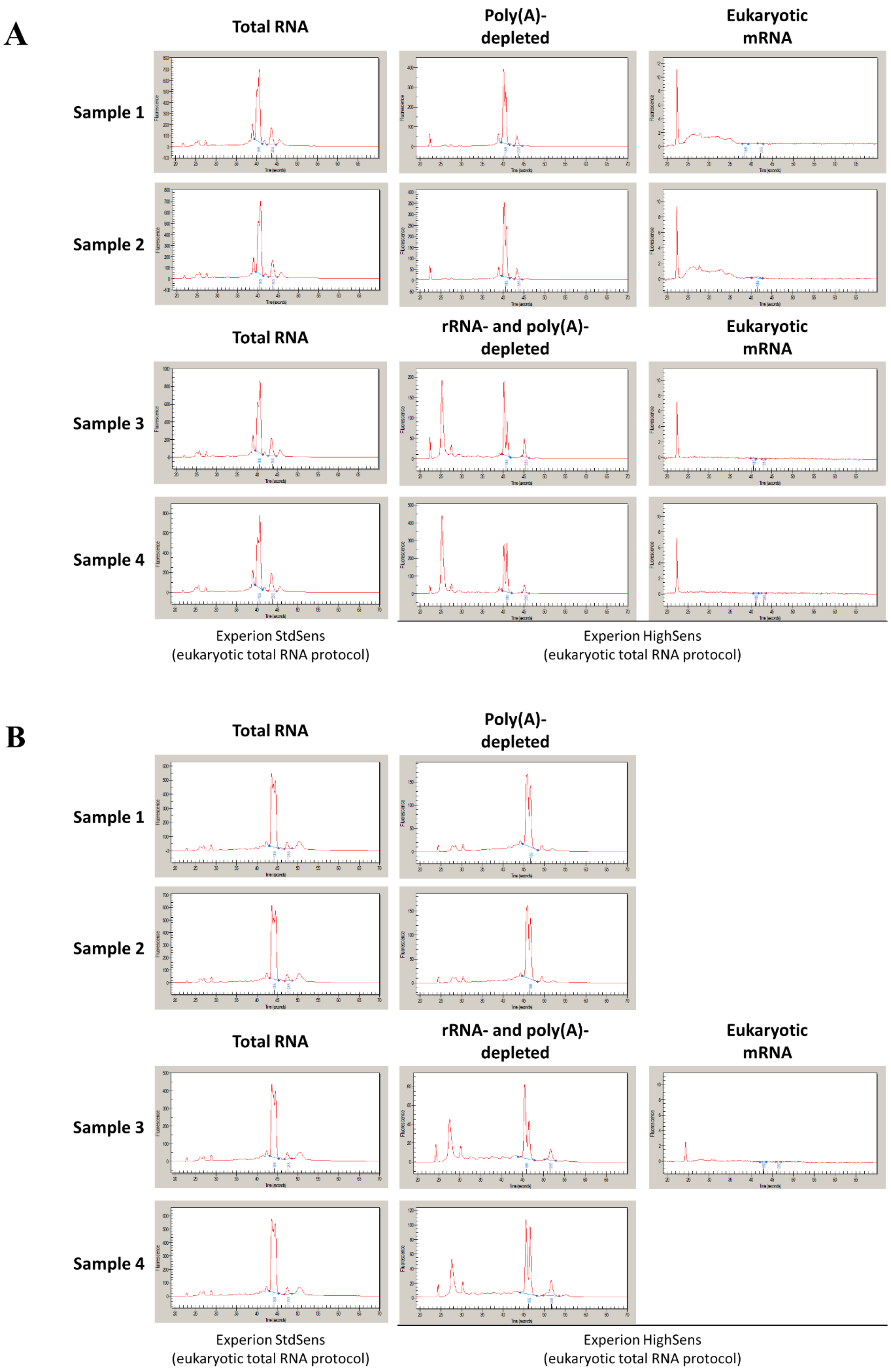
A
Total RNA
Poly(A)-
depleted
Eukaryotic
mRNA
Sample 1
Sample 2
Total RNA
rRNA- and poly(A)-
depleted
Eukaryotic
mRNA
Sample 3
Sample 4
Experion StdSens
(eukaryotic total RNA protocol)
Experion HighSens
(eukaryotic total RNA protocol)
B
Total RNA
Poly(A)-
depleted
Sample 1
Sample 2
Total RNA
rRNA- and poly(A)-
depleted
Eukaryotic
mRNA
Sample 3
Sample 4
Experion StdSens
(eukaryotic total RNA protocol)
Experion HighSens
(eukaryotic total RNA protocol)

Supplementary Figure 5. Electropherograms of poly(A)-depleted and rRNA- and poly(A)-depleted RNA. Total RNA was extracted with the miRNeasy Mini Kit from two cell culture flasks of different passages (**A, B**) in 4 technical replicates each. Poly(A) depletion was performed with Dynabeads. rRNA depletion was performed using a 1:4 ratio of Pan-Bacteria to *Aedes albopictus* riboPOOLs. Afterwards, poly(A) depletion was performed with Dynabeads. mRNA was recovered as a control and analyzed for the shown samples. All RNA types were subsequently analyzed on the Experion Automated Electrophoresis Station. Total RNA was analyzed with the Experion StdSens Kit using the eukaryotic total RNA protocol while the poly(A)-depleted RNA, rRNA- and poly(A)-depleted RNA, and eukaryotic mRNA were analyzed with the Experion HighSens Kit using the eukaryotic total RNA protocol. The poly(A)-depleted RNA was diluted 1:200 (**A**) or 1:500 (**B**) and the rRNA- and poly(A)-depleted RNA was diluted 1:2 (A) or 1:10 (B). The depleted samples of **B** were sent for RNA-seq.

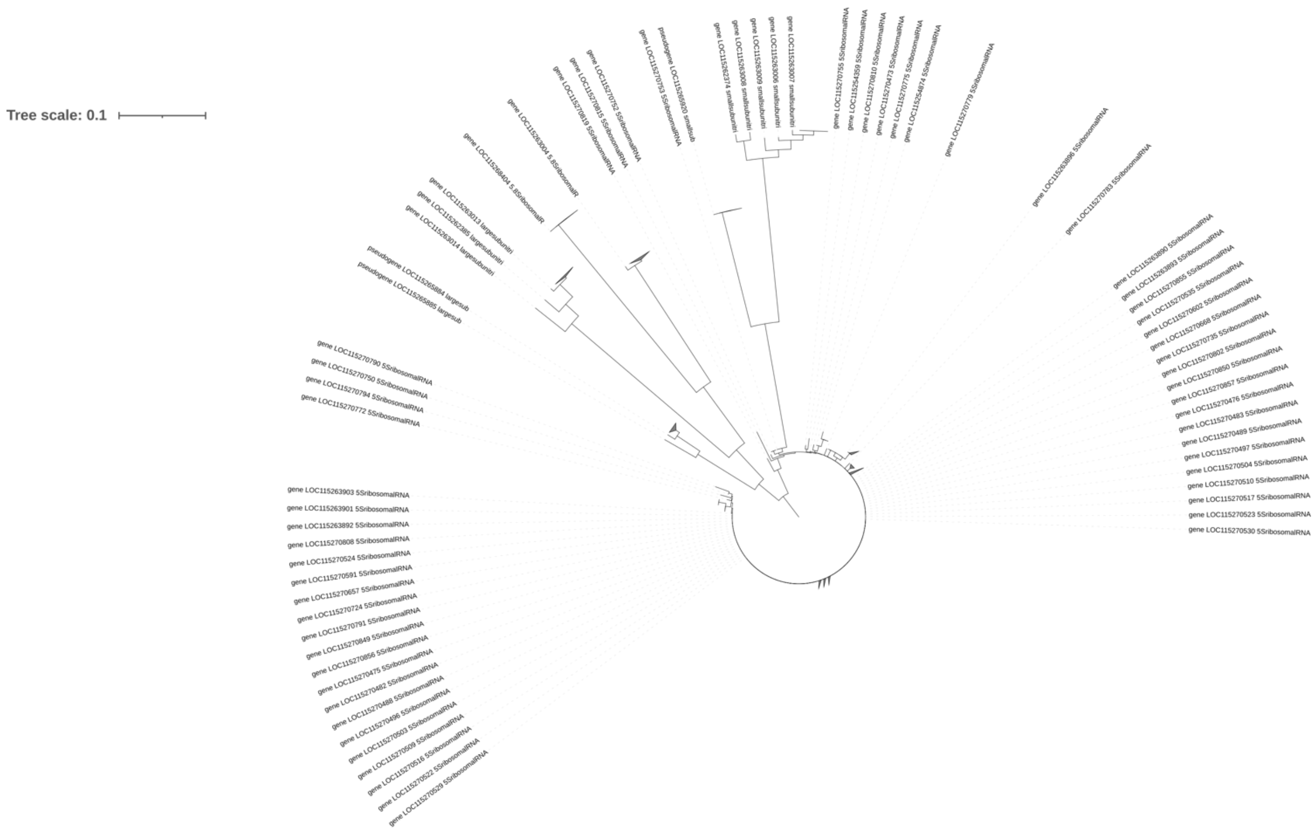

Supplementary Figure 6. *Aedes albopictus* rRNA sequences tree. A multiple sequence alignment of the rRNA genes and pseudogenes of *Aedes albopictus* (RefSeq ID: GCF_006496715.1) was created using MUSCLE (Edgar, 2004) on the EMBL-EBI sequence analysis portal (Madeira et al., 2024) and the tree was created using iTOL (Letunic and Bork, 2024). The pseudogenes showed high similarity to rRNA genes but cluster distinctly. 12 sequences were found to have less than 95% identity.

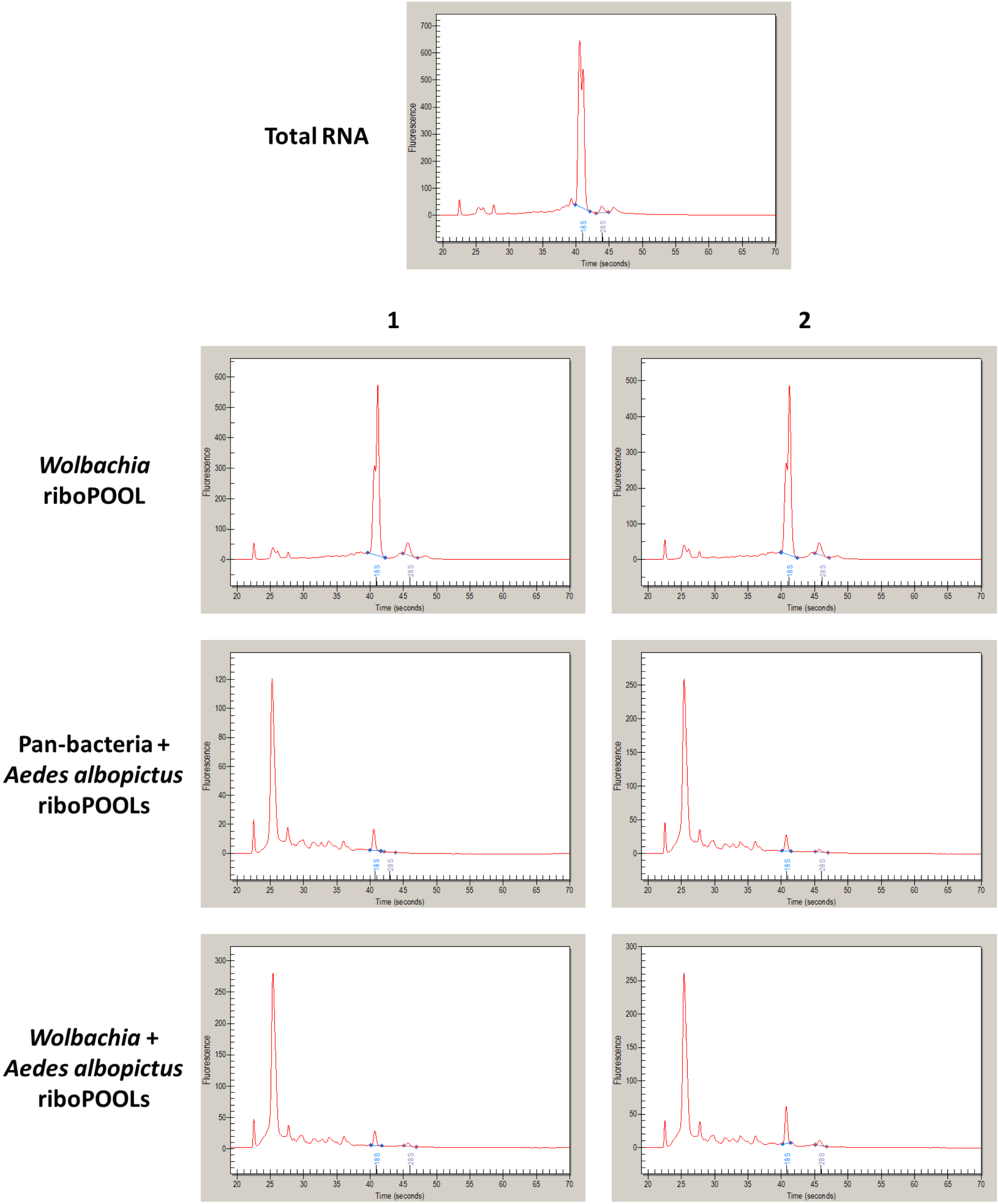

Supplementary Figure 7. Electropherograms showing depletion of wolbachial and insect cell rRNA by different riboPOOLs. Total RNA was extracted with the miRNeasy Mini Kit and subsequently treated with different riboPOOLs (1 µl *Wolbachia* riboPOOL, 2 µl of Pan-Bacteria or *Wolbachia* to *Aedes albopictus* riboPOOLs in a 1:4 ratio) in technical duplicates for rRNA depletion. Total RNA and rRNA-depleted RNA were analyzed with the Experion StdSens Kit using the eukaryotic total RNA protocol on the Experion Automated Electrophoresis Station. There was a slightly insufficient amount of the *Aedes albopictus* riboPOOL available for preparing the second replicate in combination with the *Wolbachia* riboPOOL, though the exact deficit was not quantified.

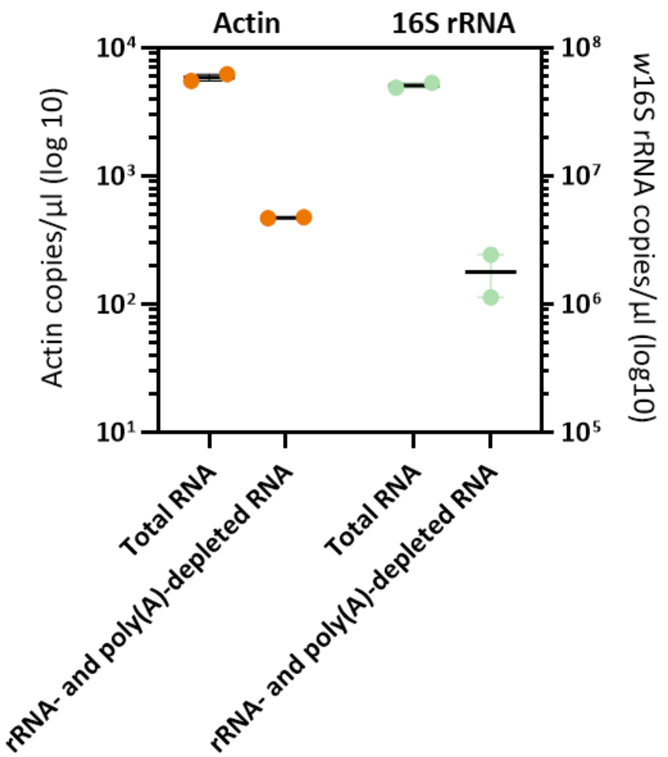

Supplementary Figure 8. Depletion of rRNA and insect cell mRNA using custom-designed riboPOOLs and Dynabeads. Total RNA was extracted with the miRNeasy Mini Kit in technical replicates, followed by rRNA depletion with riboPOOLs and subsequent poly(A) depletion. Insect cell actin (orange) and wolbachial 16S rRNA (*w*16S rRNA, green) transcripts were quantified via RT-qPCR in total RNA and rRNA- and poly(A)-depleted RNA. The mean ± SEM of two technical replicates is shown. These replicates were sent for RNA-seq.

A

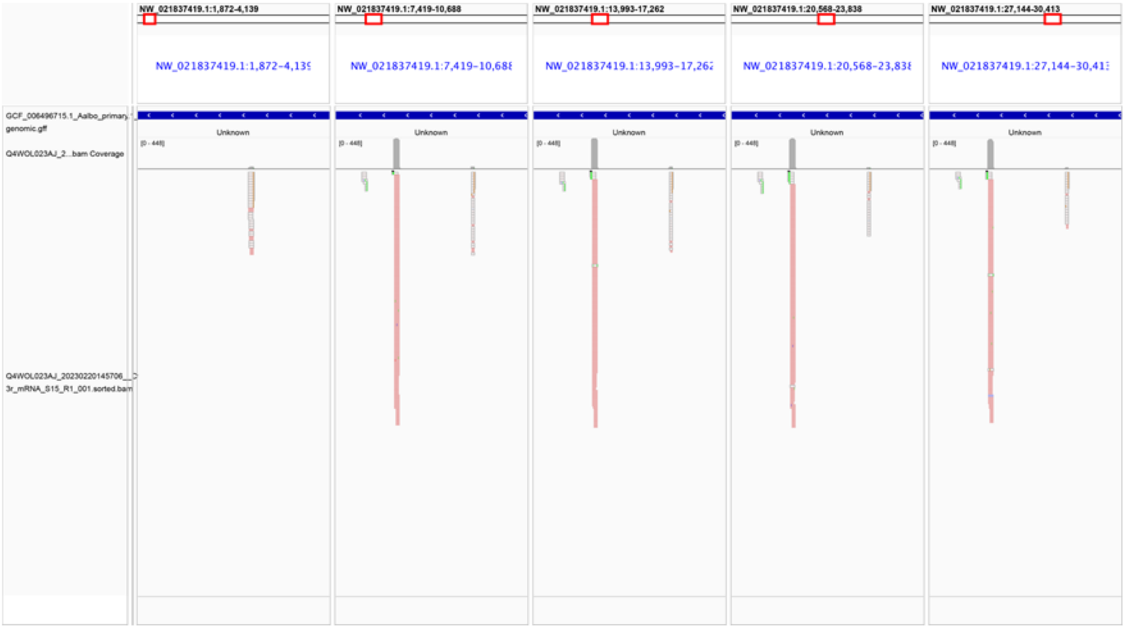
NW_021837419.1:1,872-4,139
NW_021837419.1:7,419-10,688
NW_021837419.1:13,993-17,262
NW_021837419.1:20,568-23,838
NW_021837419.1:27,144-30,413
GCF_006496715.1_Aalbo_primary.
genomic.gff
Q4WOL023AJ_2...bam Coverage
Unknown
Q4WOL023AJ_20230220145706_
3r_mRNA_S15_R1_001.sorted.bam

B

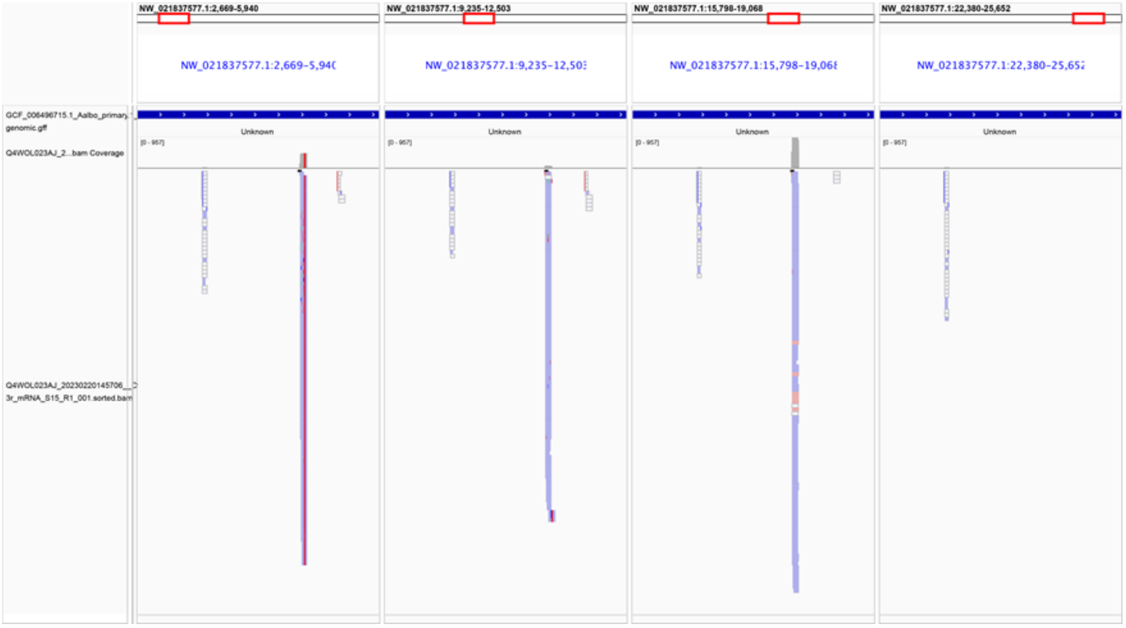
NW_021837577.1:2,669-5,940
NW_021837577.1:9,235-12,503
NW_021837577.1:15,798-19,068
NW_021837577.1:22,380-25,652
GCF_006496715.1_Aalbo_primary.
genomic.gff
Q4WOL023AJ_2...bam Coverage
Unknown
[0 - 957]
Q4WOL023AJ_20230220145706_
3r_mRNA_S15_R1_001.sorted.bam

C

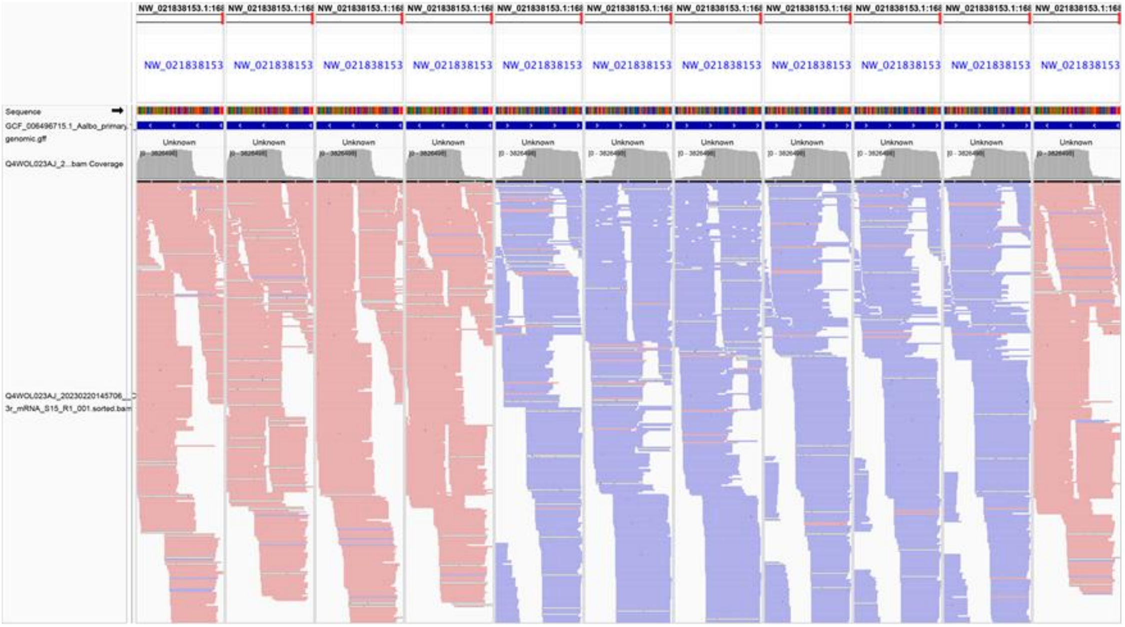

D

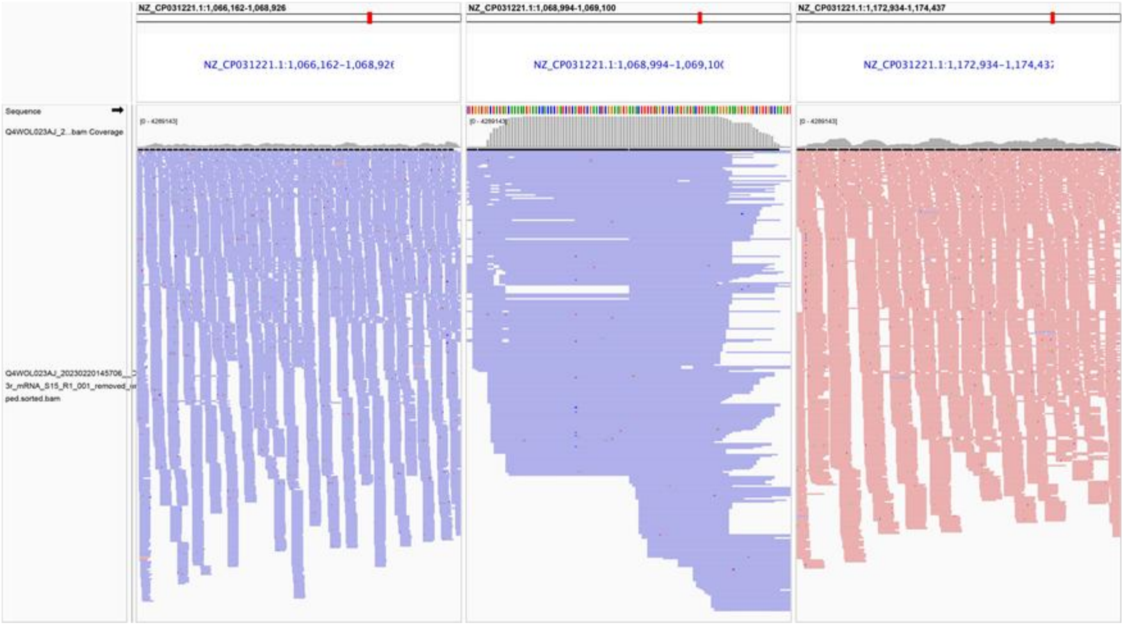

Supplementary Figure 9. IGV analysis of rRNA loci. Mapped reads were visualized using the Integrative Genomics Viewer (IGV, version 2.19.7). Representative views are shown for one of the two technical replicates for the nine annotated 28S rRNA loci (**A**, **B**) and selected 5S rRNA loci (**C**) of *Aedes albopictus* as well as the three annotated rRNA loci of *Wolbachia* (**D**).

A

B

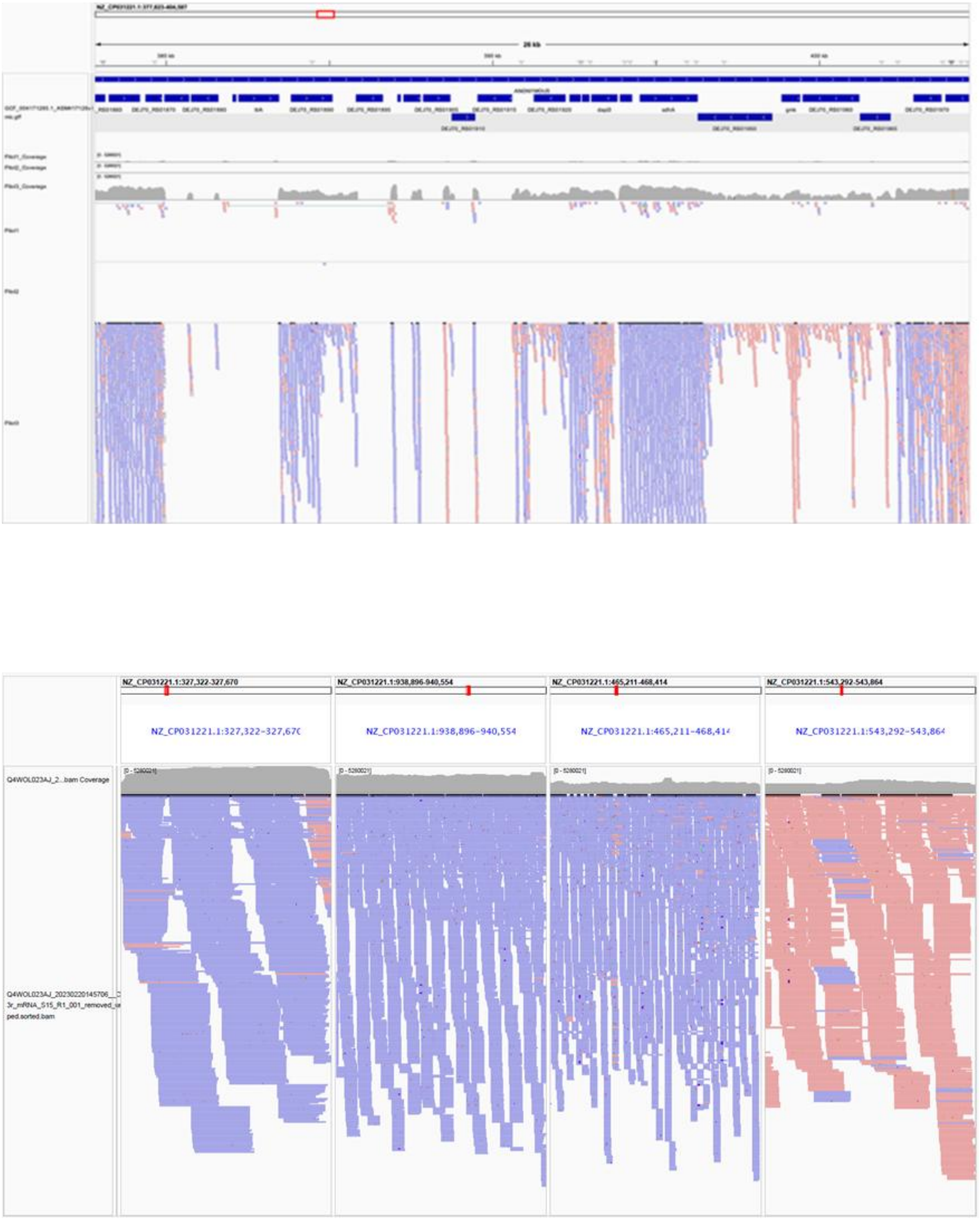

Supplementary Figure 10. IGV analysis of *Wolbachia* protein-coding genes. Mapped reads were visualized using the Integrative Genomics Viewer (IGV, version 2.19.7). Representative views are shown for one region across all three runs, with one replicate per run (**A**) and for four selected proteins for one replicate of run 3 (**B**).

Supplementary Methods

RNA extraction and quality control

To ensure high RNA integrity and yield, we optimized RNA extraction and preservation conditions. Using approximately 10^6 insect cells as starting material together with QIAzol provided the best results (Table 1, Figure 1, Supplementary Figure 1). Substituting chloroform with 1-bromo-3-chloropropane proved advantageous, as it is less toxic, requires only half the volume, and results in a more distinct phase separation (Chomczynski and Mackey, 1995).

RNA quantity and quality were assessed with the Experion Station (Bio-Rad), which has since been discontinued, as has the widely used 2100 Bioanalyzer (Agilent). Alternatives to these chip-based methods, which calculate RNA integrity numbers (RQI for Bio-Rad; RIN for Agilent), are traditional gel-based analysis of RNA integrity or other platforms for the capillary electrophoresis-based analysis, e.g., from Agilent (4150 TapeStation, 4200 TapeStation, Fragment Analyzer) or Qiagen (QIAxcel Connect). Thermo Fisher Scientific offers a method for RNA integrity determination based on fluorescent dyes (Qubit RNA IQ Assay for Qubit 4 or Qubit Flex).

Verification of depletion efficiency

To verify successful depletion, we performed RT-qPCR targeting 16S rRNA to control for bacterial rRNA depletion and, when poly(A) depletion was included, targeting actin to control for eukaryotic mRNA depletion. For adapting our method, it might be cost-effective to also include a eukaryotic rRNA RT-qPCR to the workflow, as others have done (Fauver et al., 2019; Goraichuk et al., 2024). An 18S rRNA RT-qPCR, already established for several mosquito species (Hoffmann et al., 2004), offers a way to control for eukaryotic rRNA depletion.

High-quality RNA was obtained by using at least 10^6 cells as starting material, QIAzol, the miRNeasy Mini Kit with 1-bromo-3-chloropropane, and the QIAcube automated station for RNA extraction. Under these conditions, RNA concentrations exceeded 500 ng/µl, with A260/A280 ratios of 1.9–2.0 and RQI values of 10.

Discontinued rRNA depletion kits for non-mammalian hosts

The study of intracellular bacteria in non-mammalian hosts is hampered by the lack of commercially available rRNA depletion kits. While some kits have been shown to work for non-mammalian rRNA depletion or were specifically designed for this purpose, availability has been a recurring issue. For example, the MICROBEnrich insect/*C. elegans* module (Ambion) is no longer available (Kumar et al., 2012). However, Thermo Fisher Scientific still offers the MICROBEnrich Kit for depletion of mammalian rRNA and can provide sequences from the discontinued modules for ordering oligos independently (personal communication: Thermo Fisher Scientific Tech Support). This approach has been successfully applied for insect rRNA depletion (Scully et al., 2013).

After the discontinuation of the insect module, Kumar et al. (2012) reported that the Ribo-Zero Kit (Human/Mouse/Rat) (Epicentre) efficiently removes over 98% of insect rRNA. This kit, later marketed by Illumina, was used alongside the Ribo-Zero Kit (Bacteria) in two RNA-seq studies of *Wolbachia* (Kumar et al., 2016; Chung et al., 2019). However, both kits were discontinued in 2018, further constraining available options. Similarly, the Ribo-Zero Magnetic Gold Kit (Human/Mouse/Rat) (Illumina), which also targets mitochondrial rRNA, was used in two RNA-seq studies of *Wolbachia* (Rainey et al., 2016; Leitner et al., 2021). Using this kit, Leitner et al. (2021) achieved 29.7% reads for *w*AlbB in an *Aedes aegypti* cell line, whereas Rainey et al. (2016) achieved 4.9% reads for *w*Mel in a *D. melanogaster* cell line (Rainey et al., 2016; Leitner et al., 2021). The differences in the percentage of wolbachial reads between these two studies could be due to variations in multiplicity of infection and rRNA depletion efficiency across host cells. Notably, neither study reported the percentage of rRNA reads. However, the Ribo-Zero Magnetic Gold Kit is no longer available.

Chomczynski, P., and Mackey, K. (1995). Substitution of chloroform by bromo-chloropropane in the single-step method of RNA isolation. *Anal Biochem* 225(1), 163-164. doi: 10.1006/abio.1995.1126.

Chung, M., Teigen, L.E., Libro, S., Bromley, R.E., Olley, D., Kumar, N., et al. (2019). Drug repurposing of bromodomain inhibitors as potential novel therapeutic leads for lymphatic filariasis guided by multispecies transcriptomics. *mSystems* 4(6). doi: 10.1128/mSystems.00596-19.

Edgar, R.C. (2004). MUSCLE: multiple sequence alignment with high accuracy and high throughput. *Nucleic Acids Res* 32(5), 1792-1797. doi: 10.1093/nar/gkh340.

Fauver, J.R., Akter, S., Morales, A.I.O., Black, W.C.t., Rodriguez, A.D., Stenglein, M.D., et al. (2019). A reverse-transcription/RNase H based protocol for depletion of mosquito ribosomal RNA facilitates viral intrahost evolution analysis, transcriptomics and pathogen discovery. *Virology* 528, 181-197. doi: 10.1016/j.virol.2018.12.020.

Goraichuk, I.V., Harden, M., Spackman, E., and Suarez, D.L. (2024). The 28S rRNA RT-qPCR assay for host depletion evaluation to enhance avian virus detection in Illumina and Nanopore sequencing. *Front Microbiol* 15, 1328987. doi: 10.3389/fmicb.2024.1328987.

Hoffmann, P.R., Woodrow, R.J., Calimlim, P.S., Sciulli, R., Effler, P.V., Miyamoto, V., et al. (2004). West Nile virus surveillance: a simple method for verifying the integrity of RNA in mosquito (Diptera: Culicidae) pools. *J Med Entomol* 41(4), 731-735. doi: 10.1603/0022-2585-41.4.731.

Kumar, N., Creasy, T., Sun, Y., Flowers, M., Tallon, L.J., and Dunning Hotopp, J.C. (2012). Efficient subtraction of insect rRNA prior to transcriptome analysis of *Wolbachia-Drosophila* lateral gene transfer. *BMC Res Notes* 5, 230. doi: 10.1186/1756-0500-5-230.

Kumar, N., Lin, M., Zhao, X., Ott, S., Santana-Cruz, I., Daugherty, S., et al. (2016). Efficient enrichment of bacterial mRNA from host-bacteria total RNA samples. *Sci Rep* 6, 34850. doi: 10.1038/srep34850.

Leitner, M., Bishop, C., and Asgari, S. (2021). Transcriptional response of *Wolbachia* to dengue virus infection in cells of the mosquito *Aedes aegypti*. *mSphere* 6(3)**,** e0043321. doi: 10.1128/mSphere.00433-21.

Letunic, I., and Bork, P. (2024). Interactive Tree of Life (iTOL) v6: recent updates to the phylogenetic tree display and annotation tool. *Nucleic Acids Res* 52(W1)**,** W78-w82. doi: 10.1093/nar/gkae268.

Madeira, F., Madhusoodanan, N., Lee, J., Eusebi, A., Niewielska, A., Tivey, A.R.N., et al. (2024). The EMBL-EBI Job Dispatcher sequence analysis tools framework in 2024. *Nucleic Acids Research* 52(W1)**,** W521-W525. doi: 10.1093/nar/gkae241.

Rainey, S.M., Martinez, J., McFarlane, M., Juneja, P., Sarkies, P., Lulla, A., et al. (2016). *Wolbachia* blocks viral genome replication early in infection without a transcriptional response by the endosymbiont or host small RNA pathways. *PLoS Pathog* 12(4)**,** e1005536. doi: 10.1371/journal.ppat.1005536.

Scully, E.D., Hoover, K., Carlson, J.E., Tien, M., and Geib, S.M. (2013). Midgut transcriptome profiling of *Anoplophora glabripennis*, a lignocellulose degrading cerambycid beetle. *BMC Genomics* 14(1)**,** 850. doi: 10.1186/1471-2164-14-850.
